# Ameliorative effects of melatonin on dark-induced leaf senescence in gardenia (*Gardenia jasminoides* Ellis): leaf morphology, anatomy, physiology and transcriptome

**DOI:** 10.1038/s41598-017-10799-9

**Published:** 2017-09-05

**Authors:** Daqiu Zhao, Rong Wang, Jiasong Meng, Zhiyuan Li, Yanqing Wu, Jun Tao

**Affiliations:** Jiangsu Key Laboratory of Crop Genetics and Physiology, College of Horticulture and Plant Protection, Yangzhou University, Yangzhou, China

## Abstract

Cut gardenia (*Gardenia jasminoides* Ellis) foliage is widely used as a vase material or flower bouquet indoors; however, insufficient indoor light accelerates its senescence, which shortens its viewing time. In this study, applying melatonin to delay gardenia leaf senescence when exposed to extremely low light condition (darkness), and the results showed that 1.0 mM was the effective concentration. At this concentration, chlorophyll contents and chlorophyll fluorescence parameters (F_v_/F_m_, F_v_/F_0_ and Y(II)) increased, while the carotenoid and flavonoid contents decreased. Meanwhile, stress physiological indices decreased in response to exogenous melatonin application, whereas an increase in glutamine synthetase activity, water and soluble protein contents was observed. Moreover, exogenous melatonin application also reduced leaf programmed cell death under darkness, increased the endogenous melatonin level, expression levels of tryptophan decarboxylase gene, superoxide dismutase and catalase activities and the ascorbate–glutathione cycle, and maintained more intact anatomical structures. Furthermore, transcriptome sequencing revealed that various biological processes responded to exogenous melatonin application, including carbohydrate metabolism, amino acid metabolism, lipid metabolism, plant hormone signal transduction and pigment biosynthesis. Consequently, dark-induced leaf senescence in gardenia was significantly delayed. These results provided a better understanding for improving the ornamental value of cut gardenia foliage using melatonin.

## Introduction

Melatonin is an important indoleamine, and its chemical name is *N*-acetyl-5-methoxytryptamine^1^. Since first being detected in plants in 1995^2, 3^, melatonin has been reported to be universally present in plants and exhibit a wide variety of levels^4, 5^. Subsequently, the biosynthetic pathway and physiological function of melatonin in plants have been widely studied. Many studies have demonstrated that melatonin’s biosynthetic pathway can be divided into the following four steps^6–8^: firstly, tryptophan is converted to tryptamine by tryptophan decarboxylase (TDC, E.C. 4.1.1.28); subsequently, tryptophan 5-hydroxylase (T5H, E.C. 1.14.16.4) hydroxylates the C-5 position of tryptamine to form serotonin; next, *N*-acetylserotonin by serotonin *N*-acetyltransferase (SANT, E.C. 2.3.1.87) catalyses the conversion of serotonin into *N*-acetylserotonin; and finally, hydroxyindole-*O*-methytransferase (HIOMT, E.C. 2.1.1.4) is the enzyme responsible for the final formation of melatonin. Melatonin has important roles in plant development, including regulating seed germination^9^, root growth^9–11^, flowering^12^ and leaf senescence^13^. Furthermore, melatonin has an important role in plant stress defence, such as improving resistance to low and high temperatures^14, 15^, enhancing salt tolerance^13, 16^, alleviating damage from heavy metals^17–19^, protecting plants from UV-B radiation^20^, improving defences against pathogen infection^21, 22^, among others.. To the best of our knowledge, these studies have been widely conducted in vegetables, fruits and deciduous plants, such as cucumber (*Cucumis sativus* L.)^9, 11, 15^, tomato (*Lycopersicon esculentum* Mill.)^18, 19^, cabbage (*Brassica oleracea* L. var. *capitata* L.)^17, 23^, apple (*Malus domestica* Borkh.)^21, 24, 25^, grape (*Vitis vinifera* L.)^26^ and cherry (*Prunus avium* L.)^27^. However, there were few studies of melatonin in ornamental and evergreen plants.

Gardenia (*Gardenia jasminoides* Ellis) is an evergreen small shrub that belongs to the family Rubiaceae. It originates in central China, has a cultivation history of more than 2000 years and is now widely cultivated in some Asian countries and regions because of its lush branches and leaves as well as its strongly fragrant pure white flowers^28^. With increased interest in the ornamental gardenia plant, it is now used in a landscaping function, including as a flower hedge and bonsai^29^. In recent years, the constantly improving standard of living and increasing requirements for indoor environments have given rise to an indoor greening industry and especially an increasing demand for cut flowers and cut foliage. Cut gardenia foliage is greatly favoured due to its shiny, dark green, leathery leaves with elegant fragrance and variable shape. However, whether to fill a vase alone or as the choice material in a flower bouquet, insufficient indoor light greatly accelerates its senescence, which tremendously shortens its viewing time and seriously affects its further application^30, 31^. Little is known about the relevant solutions; therefore, taking measures to delay leaf senescence under low light conditions requires investigation.

Leaf senescence due to exposure to low light conditions is a typical symptom of plants, and is regulated by plant growth regulators^32^. Several independent studies have all demonstrated that melatonin can delay leaf senescence under extremely low light condition (darkness); these studies mainly concentrated on physiological and biochemical responses^13, 23, 24, 33, 34^, different regulatory pathways have been found in different plant species^23, 24^. However, the underlying molecular mechanisms of melatonin regulation of dark-induced leaf senescence remains poorly understood. Recently, the development of transcriptome sequencing (RNA-Seq) technology has provided a highly efficient molecular biology research method^35^, which has successfully clarified the molecular mechanisms in gardenia with little genomic sequence information^29, 36^. The objectives of this study were as follows: to determine whether gardenia leaf senescence under low light conditions can be delayed by exogenous melatonin; if so, the pathway by which the effect is regulated and the underlying molecular mechanism. In this study, excised gardenia leaves were firstly treated with a melatonin solution (0, 0.05, 0.1, 1.0 and 2.0 mM) under extremely low light conditions (darkness), and the effects of different melatonin concentrations on leaf senescence were observed. Based on the optimal concentration, their physiological indices and microstructure were firstly measured and observed; subsequently, RNA-Seq was performed, and their related metabolism pathways and the expression patterns of key genes were investigated. These results could provide a theoretical basis for improving the ornamental value of cut gardenia foliage using exogenous melatonin.

## Results

### Effects of melatonin with different concentrations on dark-induced leaf senescence

**Figure 1:**
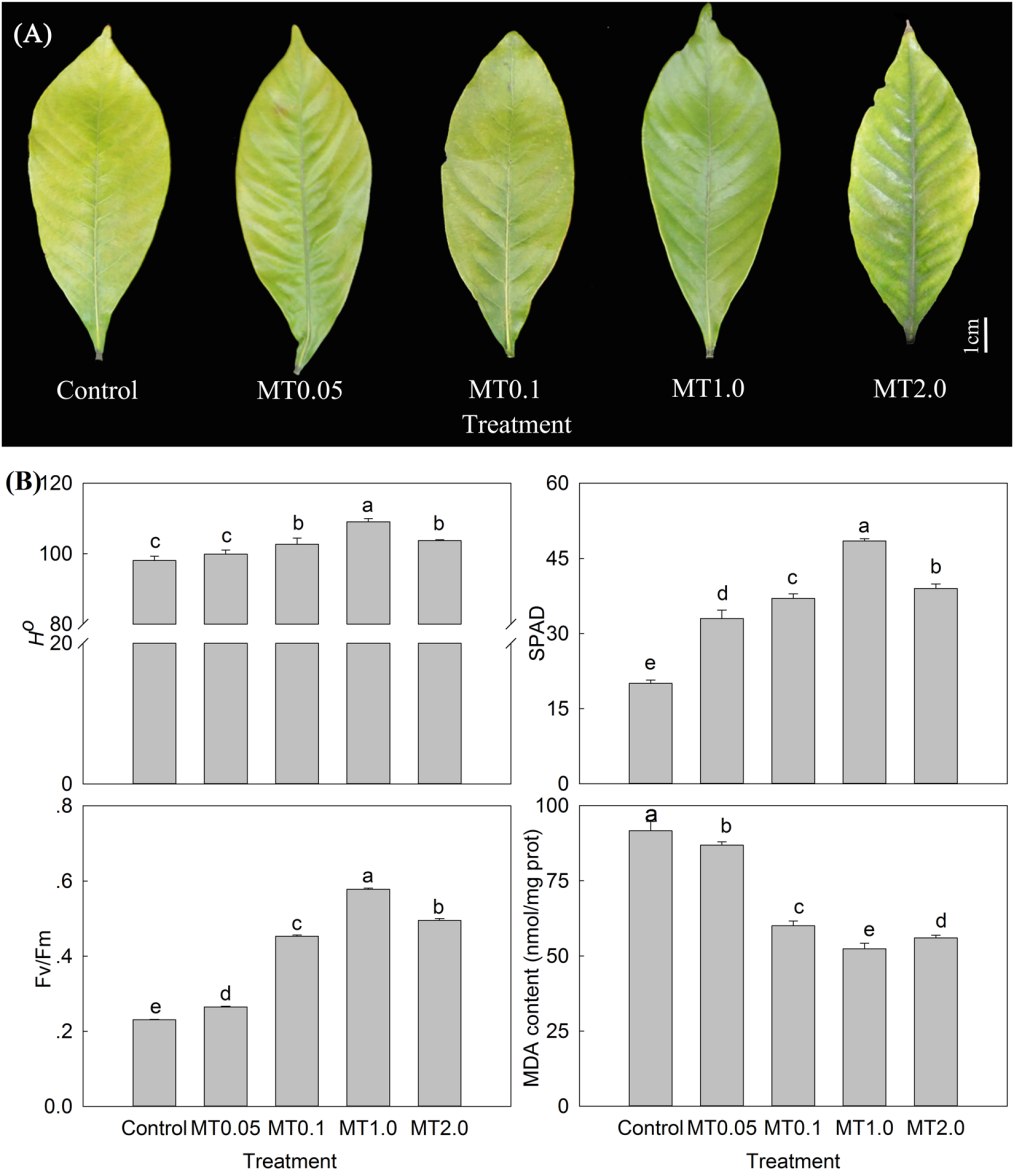
Effects of melatonin with different concentrations on dark-induced gardenia leaf senescence on day 18 after treatment. **(A)** Photograph of gardenia leaves. (**B**) Color and physiological indices of gardenia leaves. The values represent the means ± SDs, and different letters indicate significant differences according to Duncan’s multiple range test (*P* < 0.05).

As shown in Fig. 1A, four different melatonin concentrations all delayed dark-induced leaf senescence compared with the control, and 1.0 mM melatonin concentration (MT1.0)-treated leaves maintained their green color, whereas a higher concentration (2.0 mM, MT2.0) attenuated the effect. Moreover, the related color and physiological indices, including the hue angle (*H* ^*o*^), soil plant analysis development (SPAD) and the ratio of variable fluorescence to maximum fluorescence (F_v_/F_m_), all first increased and then decreased, peaking at MT1.0. Furthermore, the different treatments exhibited significant differences. Conversely, the malondialdehyde (MDA) content exhibited a significant decrease in the MT-treated leaves, and was minimized at MT1.0, MDA content for MT1.0 treatment was 52.4 nmol/mg prot, which was 42% lower than the control leaves (Fig. 1B). These indices were consistent with the visual examination, which indicated that exogenous melatonin had ameliorative effects on dark-induced gardenia leaf senescence and that MT1.0 treatment had the best effect.

### Effect of melatonin with 1.0 mM concentration on dark-induced gardenia leaf senescence during treatment

**Figure 2:**
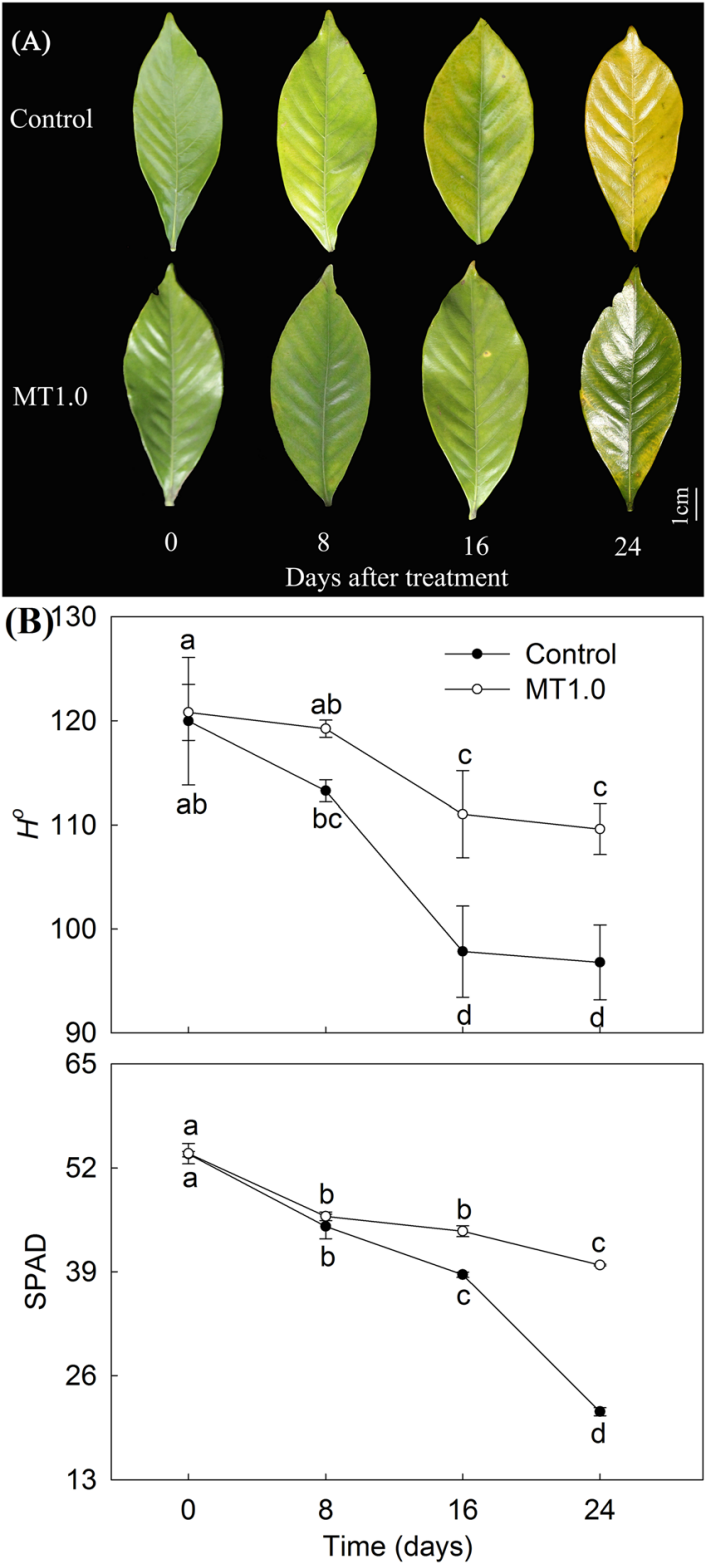
Effect of 1.0 mM melatonin on dark-induced gardenia leaf senescence during treatment. (**A**) Photograph of gardenia leaves. (**B**) Color indices of gardenia leaves. The values represent the means ± SDs, and different letters indicate significant differences according to Duncan’s multiple range test (*P* < 0.05).

Subsequently, an optimal MT1.0 effect during treatment was observed. When gardenia was transferred to continuous darkness, the leaves senesced gradually with time and were completely yellow on day 24. However, compared with the control, the progression of yellowing was slowed at MT1.0; the MT1.0-treated leaves maintained green color for a longer period, and a significant difference was observed on day 24 (Fig. 2A). The *H* ^*o*^ decreased gradually, and its value was always higher in MT1.0-treated leaves than in the control; their difference also widened gradually, especially on day 24, which showed a 13.22% increase. Similarly, the SPAD value also presented a downtrend and was higher in MT1.0-treated leaves than in the control; the biggest difference in this treatment was also observed on day 24, and the value of MT1.0-treated leaves was 1.85 times that of the control (Fig. 2B). These results were also in agreement with the visual results.

### Melatonin content and expression levels of *TDC*

**Figure 3:**
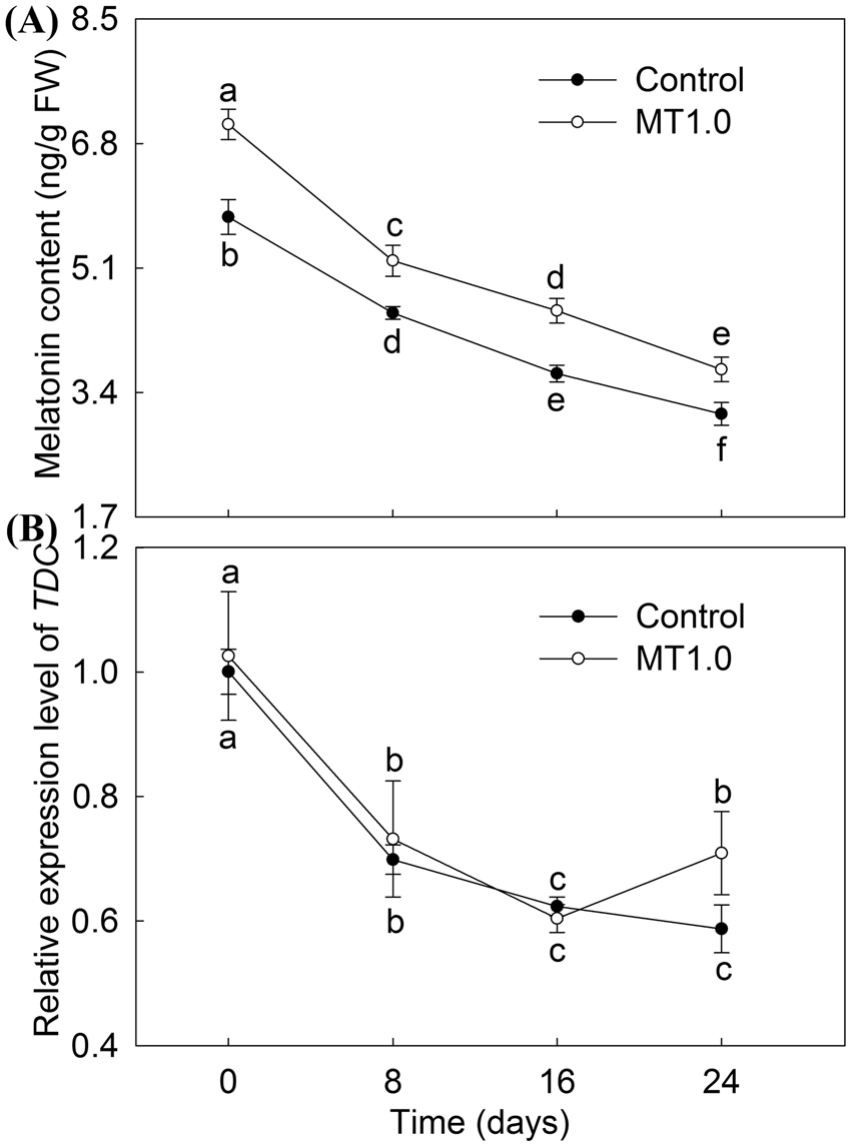
Effects of 1.0 mM melatonin on endogenous melatonin content and relative expression level of *TDC* during dark-induced gardenia leaf senescence. (**A**) Endogenous melatonin content. (**B**) Relative expression level of *TDC*. The values represent the means ± SDs, and different letters indicate significant differences according to Duncan’s multiple range test (*P* < 0.05).

The endogenous melatonin content was affected by darkness and exogenous melatonin treatment. As shown in Figs. 3A, 7.2 and 5.8 pg/g FW melatonin was detected in MT1.0-treated leaves and the control once gardenia was transferred to darkness, whereas a sharp decline of 47.38% and 46.36% was observed in MT1.0-treated leaves and the control, respectively. Moreover, the exogenous melatonin treatment significantly increased the endogenous melatonin level, and its content was higher in all MT1.0-treated leaves than in the control. Furthermore, the expression of the melatonin biosynthesis gene *TDC* was induced by exogenous melatonin treatment; it was higher in MT1.0-treated leaves than in the control, especially on day 24 (Fig. 3B).

### Pigment content and chlorophyll fluorescence parameters

**Figure 4:**
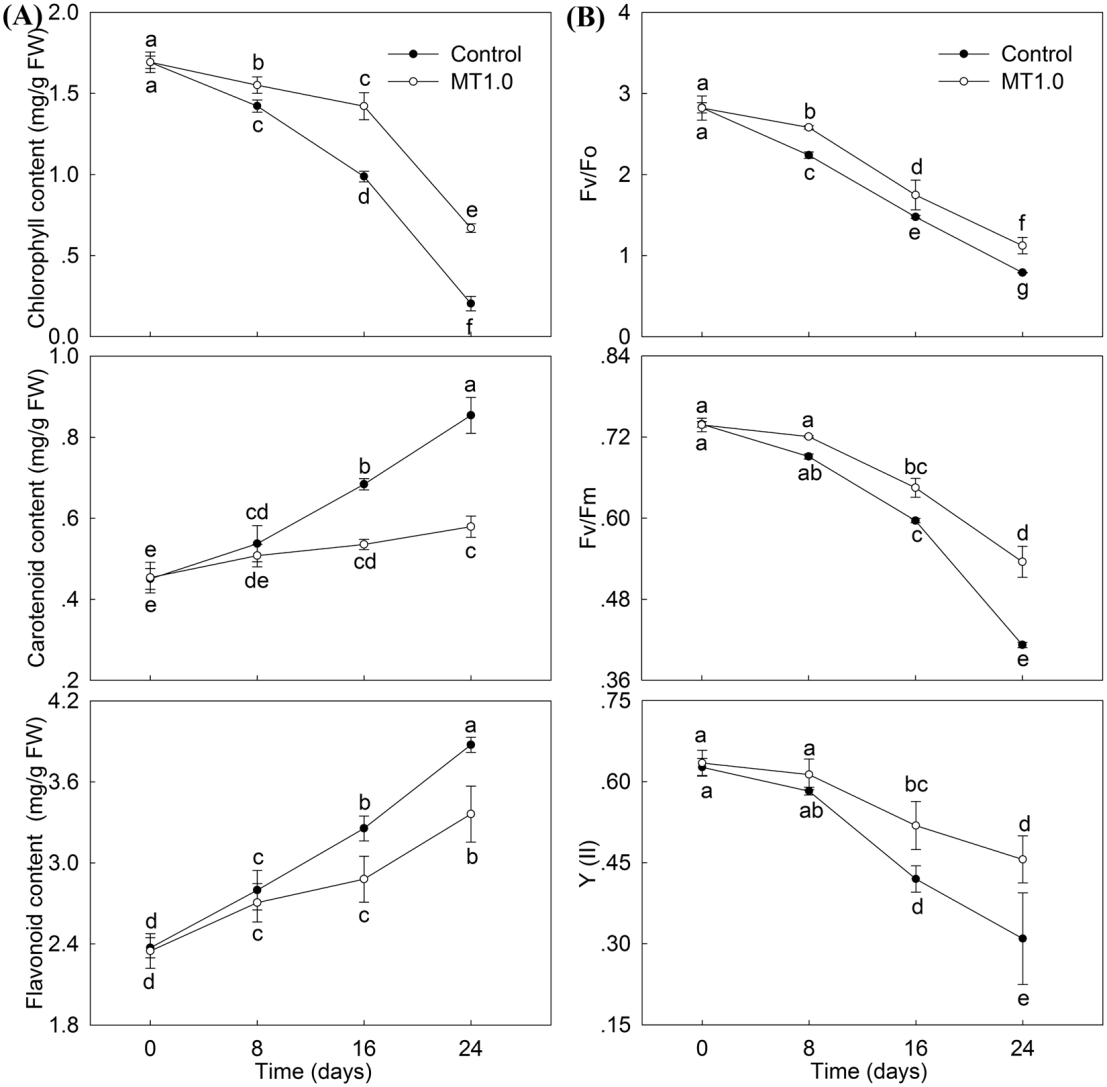
Effect of 1.0 mM melatonin on pigment contents and chlorophyll fluorescence parameters during dark-induced gardenia leaf senescence. The “FW” in the units means fresh weight. (**A**) Pigment contents. (**B**) Chlorophyll fluorescence parameters. The values represent the means ± SDs, and different letters indicate significant differences according to Duncan’s multiple range test (*P* < 0.05).

Dark-induced gardenia leaf senescence was accompanied by the pigment change. The chlorophyll contents in MT1.0-treated leaves and the control all decreased with increasing darkness, whereas the opposite trend was observed in carotenoid and flavonoid contents. In addition, MT1.0 treatment increased chlorophyll content but decreased carotenoid and flavonoid contents; their greatest differences were all observed on day 24, the chlorophyll content in MT1.0-treated leaves was 1.95 times that of the control, which indicated that MT1.0-treated leaves maintained their chlorophyll well (Fig. 4A). Subsequently, the correlation between greenness and leaf function, the ratio of variable fluorescence to minimal fluorescence reflecting the potential (F_v_/F_0_) and maximum (F_v_/F_m_) photochemical efficiency of photosystem II (PSII), and the actual photosynthetic efficiency of light system II (Y(II)) reflecting the light energy exchanging efficiency of PSII were all measured. When darkness developed, the F_v_/F_0_ decrease was evaluated till 39.84% in MT1.0-treated leaves and 28.00% in the control; similarly, F_v_/F_m_ decreased by 72.54% in MT1.0-treated leaves and 55.80% in the control, and Y(II) decreased by 71.85% in MT1.0-treated leaves and 49.36% in the control. Furthermore, F_v_/F_o_, F_v_/F_m_ and Y(II) in MT1.0-treated leaves were all higher than in the control, and significant differences were all detected on day 24 (Fig. 4B).

### Stress physiological indices

**Figure 5:**
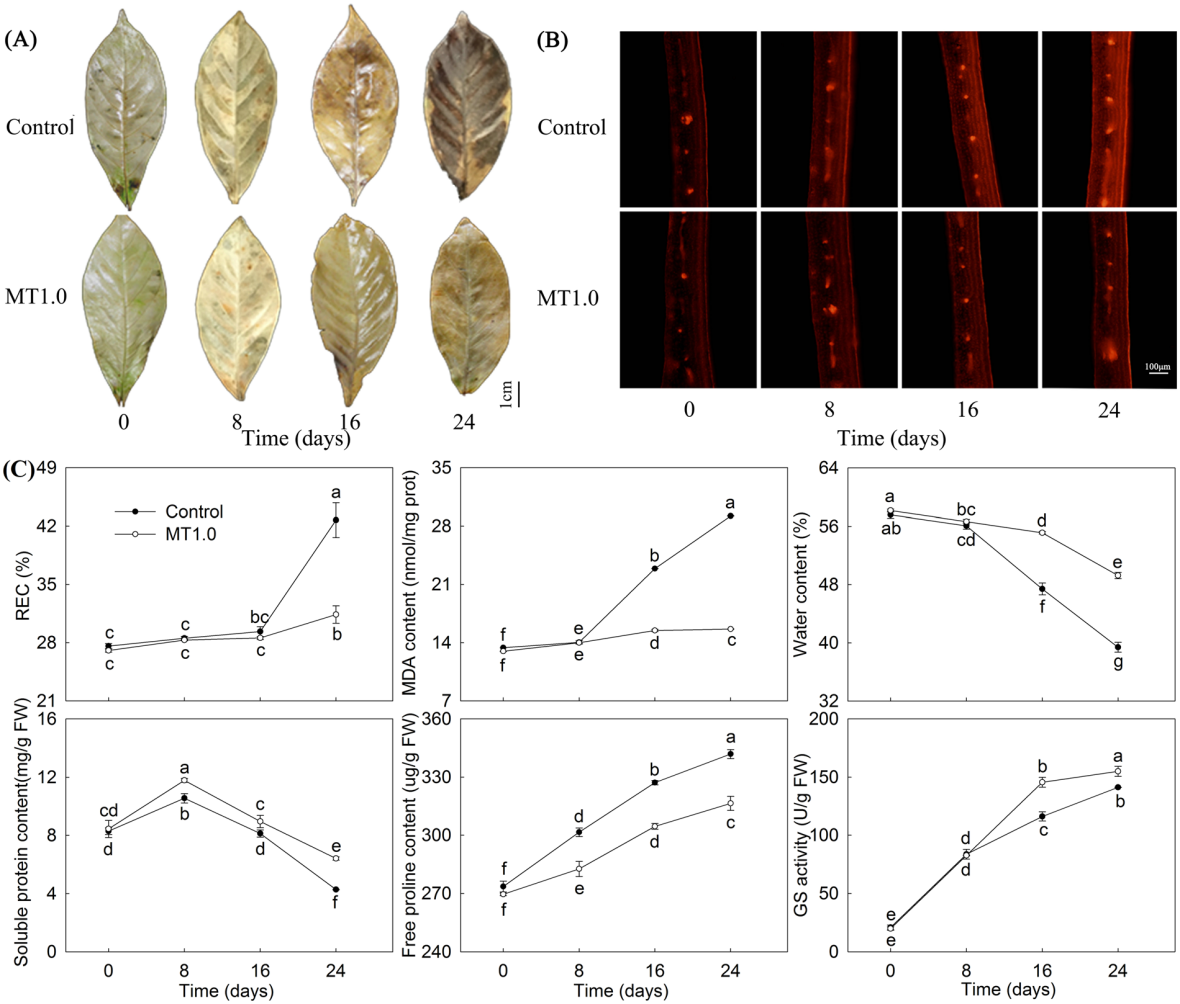
Effect of 1.0 mM melatonin on stress physiological indices during dark-induced gardenia leaf senescence. **(A)** H_2_O_2_ accumulation was detected by DAB staining. **(B)** O_2_^−^accumulation was detected by fluorescence probe. **(C)** Other physiological indices. The values represent the means ± SDs, and different letters indicate significant differences according to Duncan’s multiple range test (*P* < 0.05).

Firstly, we determined the hydrogen peroxide (H_2_O_2_) content according to diaminobezidin (DAB) staining. The results indicated that no significant differences were detected between MT1.0-treated leaves and the control on days 0 and 8, but with the development of darkness, H_2_O_2_ accumulation dramatically increased, especially on day 24 when a light color was observed on MT1.0-treated leaves compared with the control (Fig. 5A). Secondly, we detected the accumulation of superoxide anion free radical (O_2_^−^) using a fluorescence probe. The results indicated a very similar fluorescence intensity of MT1.0-treated leaves and the control after observation on days 0 and 8; then, O_2_^−^ accumulated dramatically and the fluorescence intensity of MT1.0-treated leaves was significantly lower than that of the control, especially on day 24 (Fig. 5B). Furthermore, fluorescent signals obtained using ZEN software also confirmed the above results, and the signals in the control were 1.65 times those of MT1.0-treated leaves (Supplemental Table S2). Moreover, relative electrolyte leakage (REC) and MDA levels, reflecting membrane lipid peroxidation, were determined and increased with the development of darkness; the REC content was 11.34% lower and MDA content was 46.49% lower in MT1.0-treated leaves than in the control. Additionally, other physiological indices including water, soluble protein and free proline (Pro) contents, as well as GS (EC 6.3.1.2) activity, were determined. The Pro contents and GS activity increased, whereas the water and solution protein contents decreased with the development of darkness: water, solution protein, and GS activity were higher in MT1.0-treated leaves than in the control, but Pro content was not. Significant differences in all six of this, physiological indices were detected between MT1.0-treated leaves and the control on day 24 (Fig. 5C).

**Figure 6:**
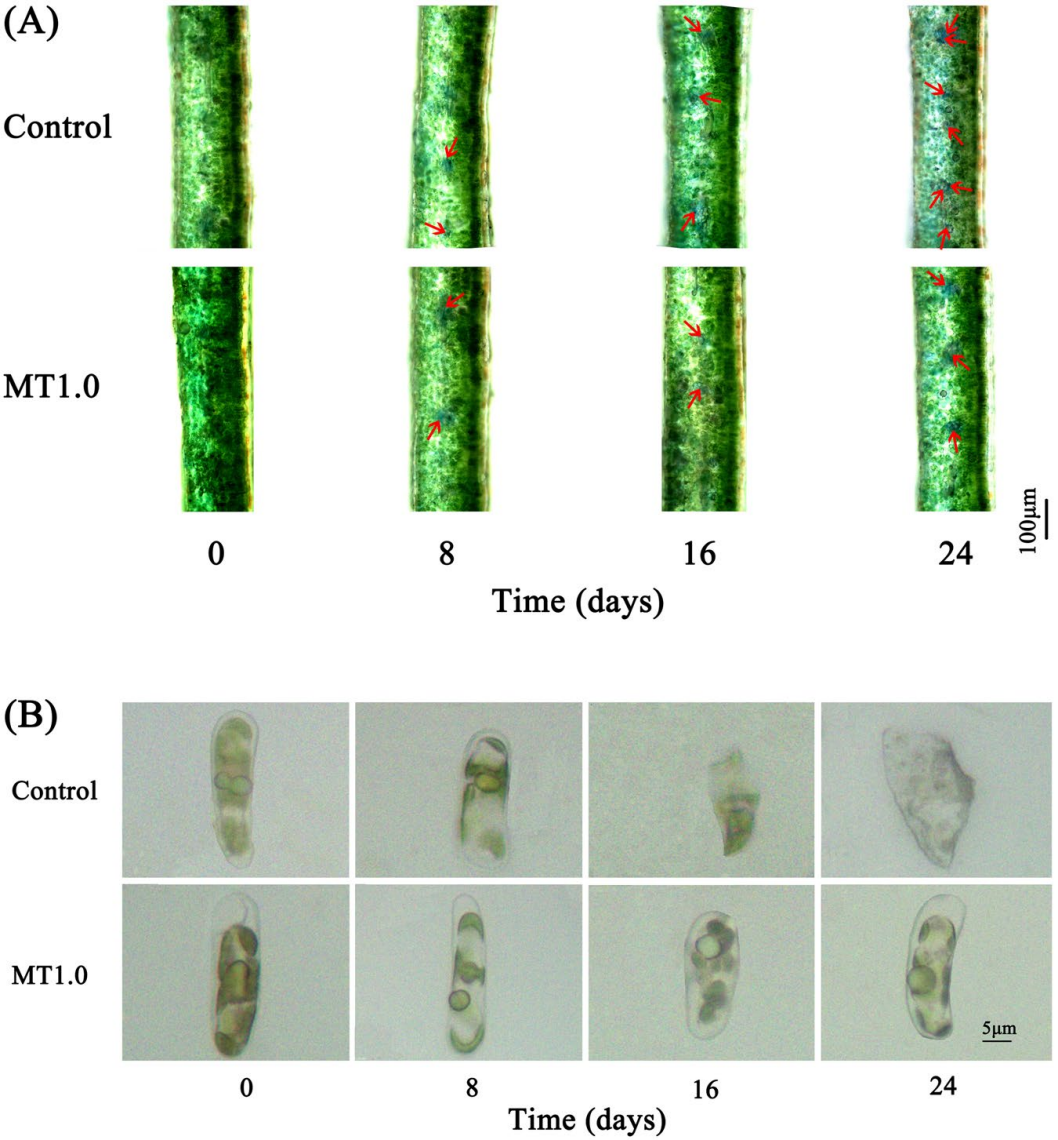
Effect of 1.0 mM melatonin on programmed cell death during dark-induced gardenia leaf senescence. **(A)** PCD detection by staining with Evans blue solution. Cell death is indicated by cells stained blue and marked by a red arrow. **(B)** PCD detection by the isolated mesophyll cell observation.

As shown in Fig. 6A, many green mesophyll cells were observed on day 0, and blue cells began to appear with the development of darkness. The highest number in the control was on day 24, whereas the fewest blue cells were detected in MT1.0-treated leaves. Meanwhile, the damaged cell membrane of isolated mesophyll cell was observed in the control, and cell nuclei disintegrated on days 18 and 24, whereas in MT1.0-treated leaves, intact cell membrane and cell membrane were detected on days 18 and 24 (Fig. 6B).

### Antioxidant enzyme activities and ascorbate–glutathione (AsA–GSH) cycle

**Figure 7:**
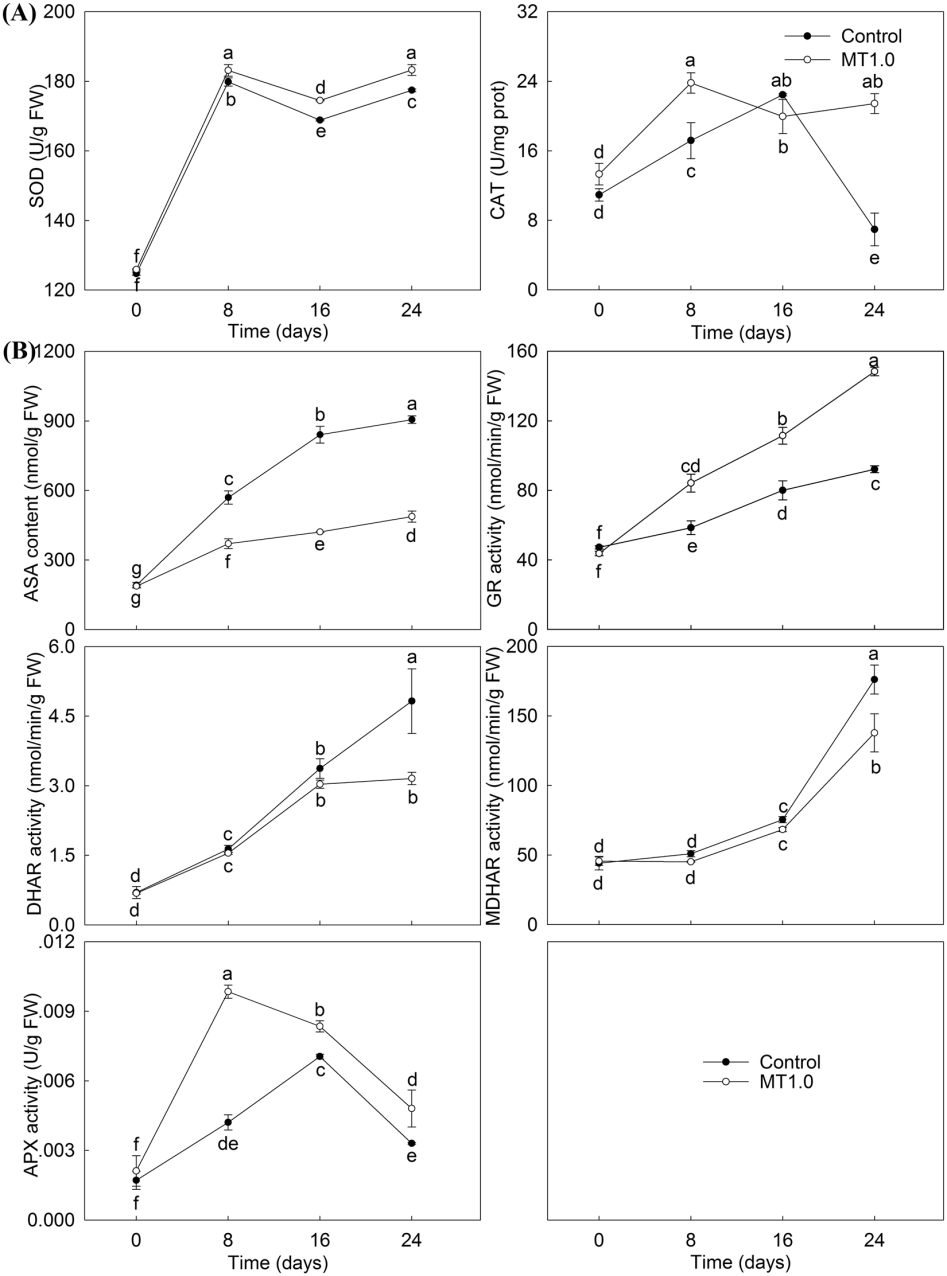
Effect of 1.0 mM melatoninon protective enzyme activities and AsA-GSH cycle during dark-induced gardenia leaf senescence. (**A**) Protective enzyme activities. (**B**) AsA-GSH cycle. The values represent the means ± SDs, and different letters indicate significant differences according to Duncan’s multiple range test (*P* < 0.05).

Antioxidant enzyme activities including superoxide dismutase (SOD, EC 1.15.1.1) and catalase (CAT, EC 1.11.1.6) were further examined. The activities of SOD and CAT increased, and they were higher in MT1.0-treated leaves than in the control; the SOD activity was especially obvious. On day 24, a significant difference in the activities of SOD and CAT was found between MT1.0-treated leaves and the control (Fig. 7A). The AsA-GSH cycle is a very important antioxidant defence system in plants and plays an important role in scavenging reactive oxygen species (ROS). As shown in Fig. 7B, the ascorbic acid (ASA) content increased with increasing darkness and was significantly lower in MT1.0-treated leaves than in the control. The largest difference reached 400.67 nmol/g FW on day 24. Moreover, an upward trend was also found in the activities of glutathione reductase (GR, EC 1.6.4.2), dehydroascorbate reductase (DHAR, EC 1.8.5.1) and monodehydroascorbate reductase (MDHAR, EC 1.6.5.4), and MT1.0-treated leaves maintained relatively higher activities of GR; nevertheless, the activities of DHAR and MDHAR markedly decreased in leaves treated with MT1.0. Additionally, the ascorbate peroxidase (APX, EC 1.11.1.11) activity first increased and then decreased, and it peaked on day 8 for MT1.0-treated leaves, and day 16 for the control; the activity in MT1.0-treated leaves was significantly higher than in the control.

### Anatomy observations

**Figure 8:**
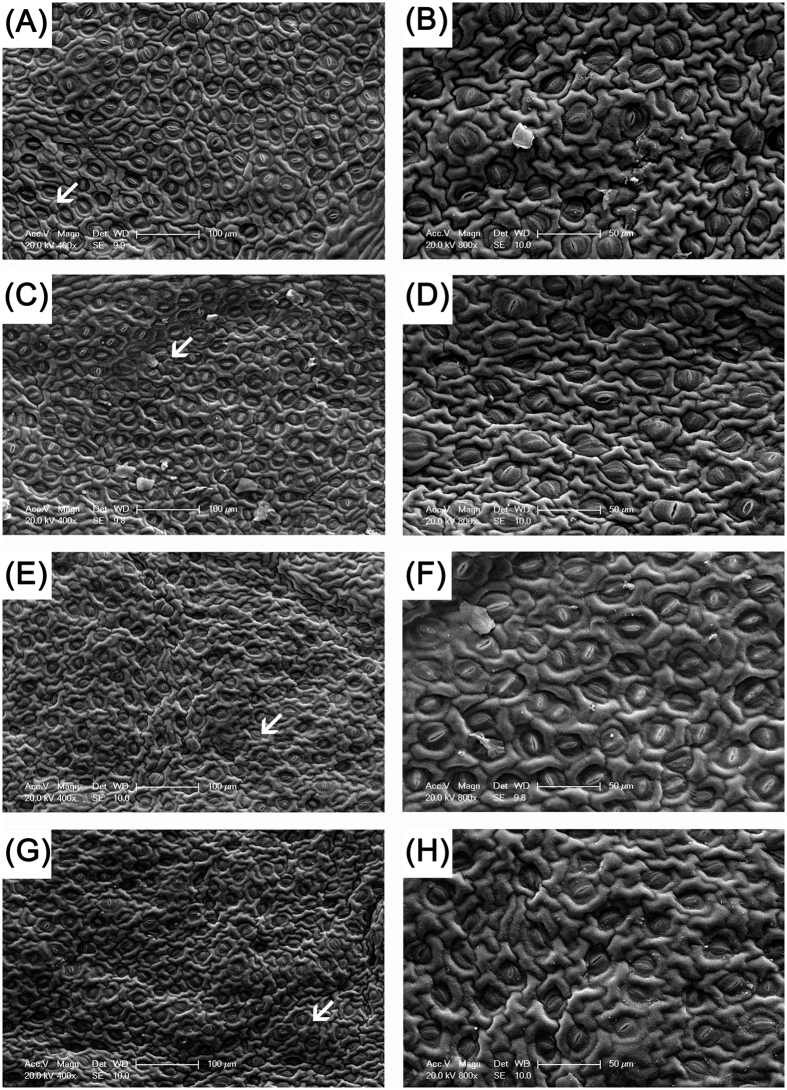
Effect of 1.0 mM melatonin on cuticle and stomatal characteristics during dark-induced gardenia leaf senescence. (**A**) Scanning electron micrograph of control on day 0. **(B)** Scanning electron micrograph of partial enlargement in **(A)** marked by an arrow. **(C)** Scanning electron micrograph of MT1.0 treatment on day 0. **(D)** Scanning electron micrograph of partial enlargement in **(C)** marked by an arrow. **(E)** Scanning electron micrograph of the control on day 24. **(F)** Scanning electron micrograph of partial enlargement in **(E)** marked by an arrow. **(G)** Scanning electron micrograph of MT1.0 treatment on day 24. **(H)** Scanning electron micrograph of partial enlargement in **(G)** marked by an arrow.

**Figure 9:**
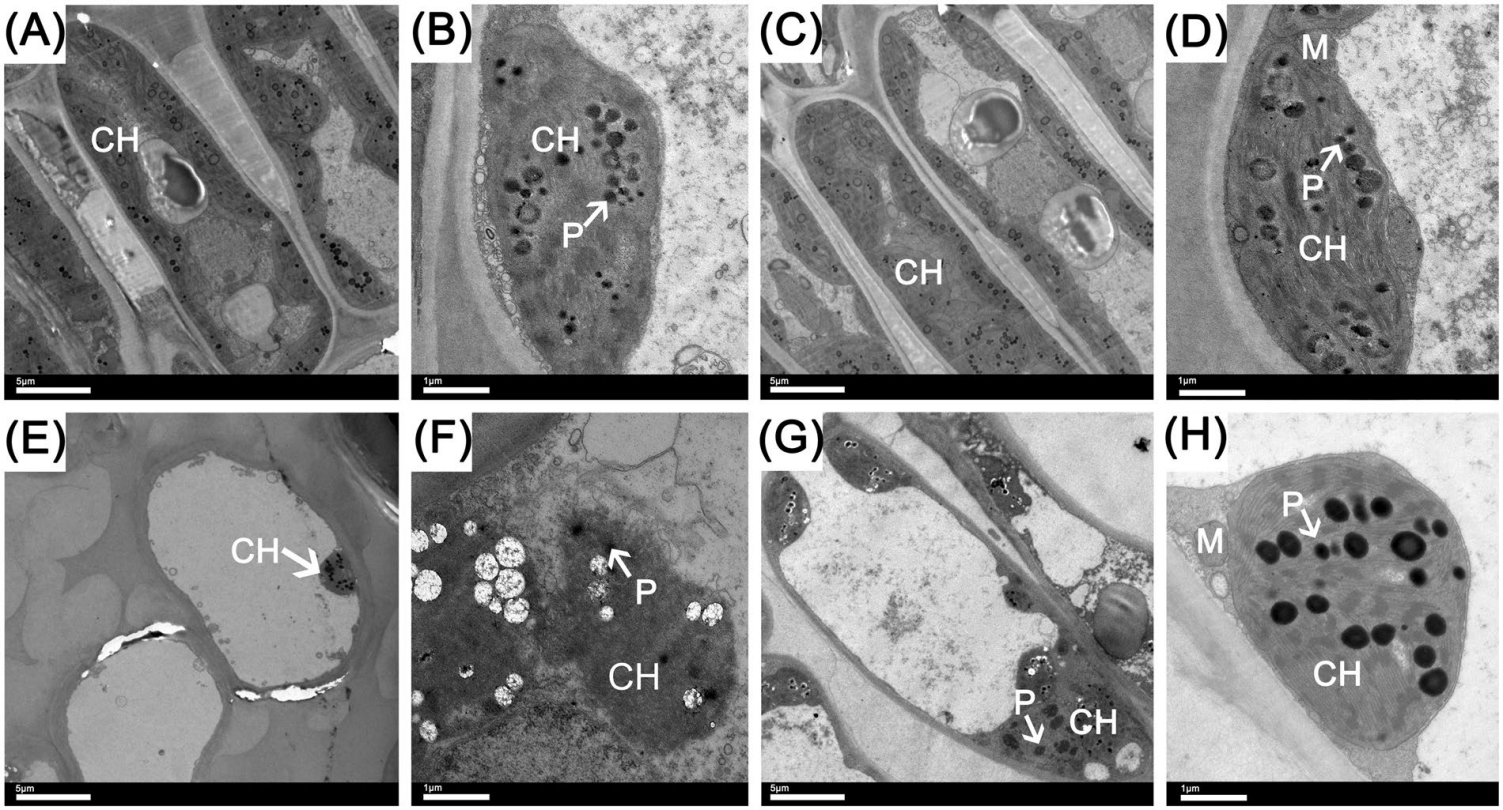
Effect of 1.0 mM melatonin on cellular ultrastructure during dark-induced gardenia leaf senescence. **(A**) Mesophyll cell of the control on day 0. **(B)** The chloroplast of the control on day 0. **(C)** Mesophyll cell of MT1.0 treatment on day 0. **(D)** Chloroplast of MT1.0 treatment on day 0. **(E)** Mesophyll cell of the control on day 24. **(F)** Chloroplast of the control on day 24. **(G)** Mesophyll cell of MT1.0 treatment on day 24. **(H)** Chloroplast of MT1.0 treatment on day 24. CH, chloroplast; P, plastoglobuli; M, mitochondrion.

The anatomical structure of leaves has an important effect on their function. In this study, the microstructure of the leaf surface was observed. On day 0, there was no obvious difference between MT1.0-treated leaves and the control. They all indicated that the cuticle of the leaf epidermis was obvious and had a corrugated texture, the long elliptic stomata were equally distributed and their density, length, width and degree of openness were also similar (Fig. 8A–D). However, on day 24, the cuticle of the leaf epidermis in MT1.0-treated leaves was still obvious and corrugated, whereas its texture in the control was flat. Regarding the density, length, width and opening degree of stomata, there were no obvious differences, but the degree of opening of stomata was higher than on day 0 (Fig. 8E–H). Subsequently, the observation and analysis of mesophyll cell ultrastructures from day 0 to day 24 indicated that the mesophyll cell ultrastructures in MT1.0-treated leaves and the control were normal and similar to each other on day 0. Chloroplasts were the most prominent cell organelle, and they were arranged close to the cell membrane in greater quantities, were mostly oval in shape and contained some bored small lipid spheres (Fig. 9A–D). On day 24, MT1.0-treated leaves had more intact mesophyll cell ultrastructures and more chloroplasts than the control. Furthermore, the chloroplasts had a more rounded shape and more lipid spheres were enlarged than previously observed. The membranes of some chloroplasts in the control appeared to be diffuse, not intact, leading to leakage of the grana lamellae (Fig. 9E–H).

### RNA-Seq and data analysis

**Figure 10:**
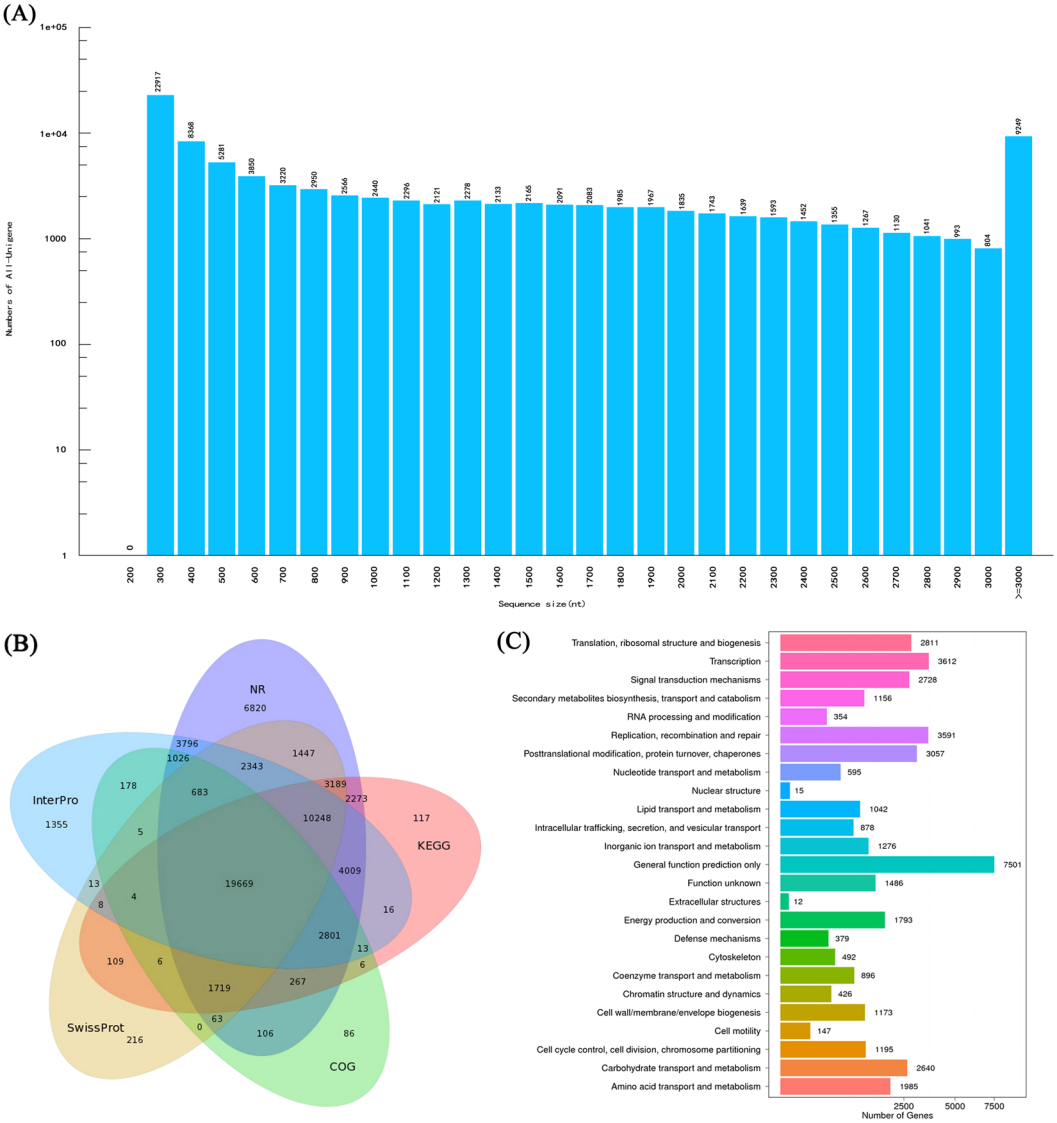
Length distribution and annotation of unigenes. **(A)** Unigene length distribution. The X axis represents the length of unigenes; the Y axis represents the number of unigenes. **(B)** Venn diagram of the number of unigenes annotated in five public databases. **(C)** Functional distribution of COG annotation. The X axis represents the number of unigenes; the Y axis represents the COG functional category.

To further elucidate the molecular mechanism of melatonin in delaying dark-induced gardenia leaf senescence, RNA-Seq was performed using the Illumina Hiseq platform. Six libraries were constructed, and 40.01 Gb of sequence data were obtained (NCBI accessions: SRP090910). After removal of the low-quality reads, the other reads were assembled into a total of 94,812 high-quality unigenes with an average length of 1,289 nt (Figure 10A, Table 1). Subsequently, these unigenes were annotated using seven public databases, including non-redundant protein database (NR), non-redundant nucleotide database (NT), gene ontology database (GO), cluster of orthologous groups of proteins database (COG), kyoto encyclopaedia of genes and genomes database (KEGG), Swiss-Prot protein database (Swiss-Prot) and Interpro. A total of 67,690 unigenes were annotated, which accounted for 71.39%. Among them, 60,459, 55,428, 46,167, 44,454, 39722 and 26,632 unigenes could be annotated to NR, NT, Interpro, KEGG, Swiss-Prot and COG databases, accounting for 63.77%, 58.46%, 48.69%, 46.89%, 41.90% and 28.09%, respectively; in the GO database, only 6,828 unigenes were annotated, which accounted for 7.20% (Fig. 10B). For the COG annotation, 26,632 unigenes were classified into 25 different functional categories, and the largest group was categorized as general function prediction only (7,501 unigenes), followed by the group with transcription (3,612 unigenes), whereas the smallest group was categorized as extracellular structures (12 unigenes) (Fig. 10C).

**Table 1:** Quality metrics of unigenes in gardenia 1.0 mM melatonin-treated leaves and the control of gardenia.

Sample	Total number	Total length (nt)	Mean length (nt)	N50	N70	N90	GC (%)
Control-1	61,668	64,265,844	1,042	1,860	1,196	393	41.74
Control-2	59,538	64,658,771	1,086	1,915	1,242	421	41.67
Control-3	58,856	64,980,805	1,104	1,941	1,271	431	41.69
MT1.0-1	59,297	62,792,525	1,058	1,850	1,207	410	41.75
MT1.0-2	62,609	67,595,057	1,079	1,911	1,241	416	41.55
MT1.0-3	58,389	62,770,884	1,075	1,853	1,228	425	41.66
All-Unigene	94,812	122,278,346	1,289	2,238	1,543	570	41.46

N50: weighted median statistic that 50% of total length is contained in unigenes greater than or equal to this value. GC (%): percentage of G and C bases in All Unigenes.

**Figure 11:**
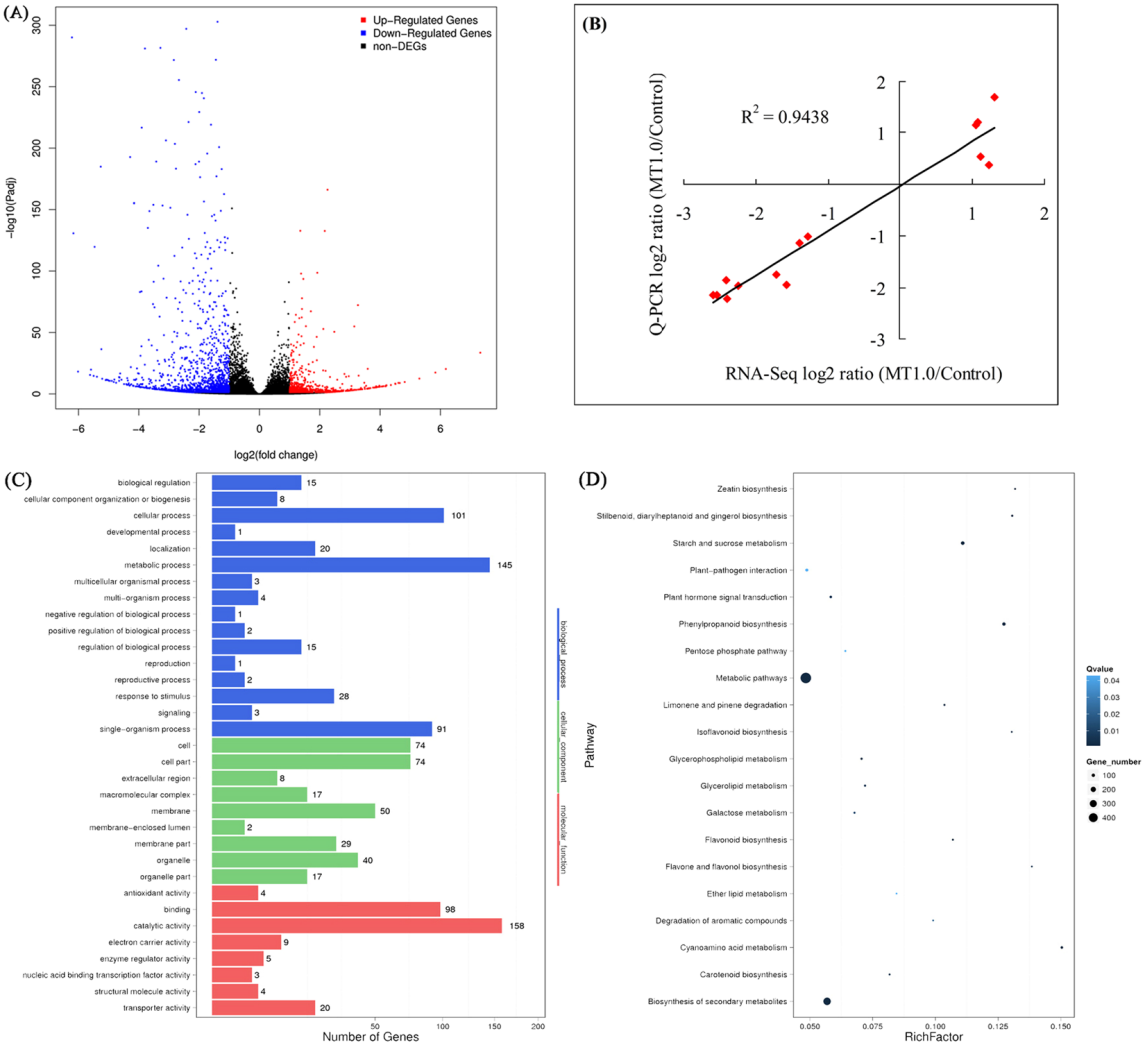
Functions and validation of DEGs. **(A**) Volcano plot of DEGs. The X axis represents log2 transformed fold change; the Y axis represents -log10 transformed adjusted P value, the red points represent up-regulated DEGs, the blue points represent down-regulated DEGs and the black points represent non-DEGs. **(B)** Correlation of gene expression results obtained from RNA-Seq (X axis) and Q-PCR (Y axis) analysis. Correlation assay performed for 14 DEGs with log2 ratio ≥1.00 or ≤−1.00. **(C)** GO classification of DEGs. The X axis represents the number of DEGs; the Y axis represents the GO term. **(D)** Pathway functional enrichment of DEGs. The X axis represents the enrichment factor, the Y axis represents the pathway name, coloring indicates q-value, a lower q-value indicates more significant enrichment, and the point size indicates the DEG number.

Furthermore, the transcriptomes of MT1.0-treated leaves and the control were comparatively analyzed to identify the differentially expressed genes (DEGs), and 2,354 DEGs based on fold change ≥2.0 and adjusted P-value ≤0.05 were obtained. Among them, the expression levels of 782 DEGs were up-regulated and those of 1,572 DEGs were down-regulated (Fig. 11A). To validate these DEGs, the expression levels of 14 randomly selected genes were analysed by real-time quantitative polymerase chain reaction (Q-PCR), and the result is shown in Fig. 11B. By comparison, a significant positive correlation (R^2^ = 0.9438) was found between the RNA-Seq and Q-PCR results, and their regression slope was close to 1.0, which revealed that the RNA-Seq results were credible.

**Figure 12:**
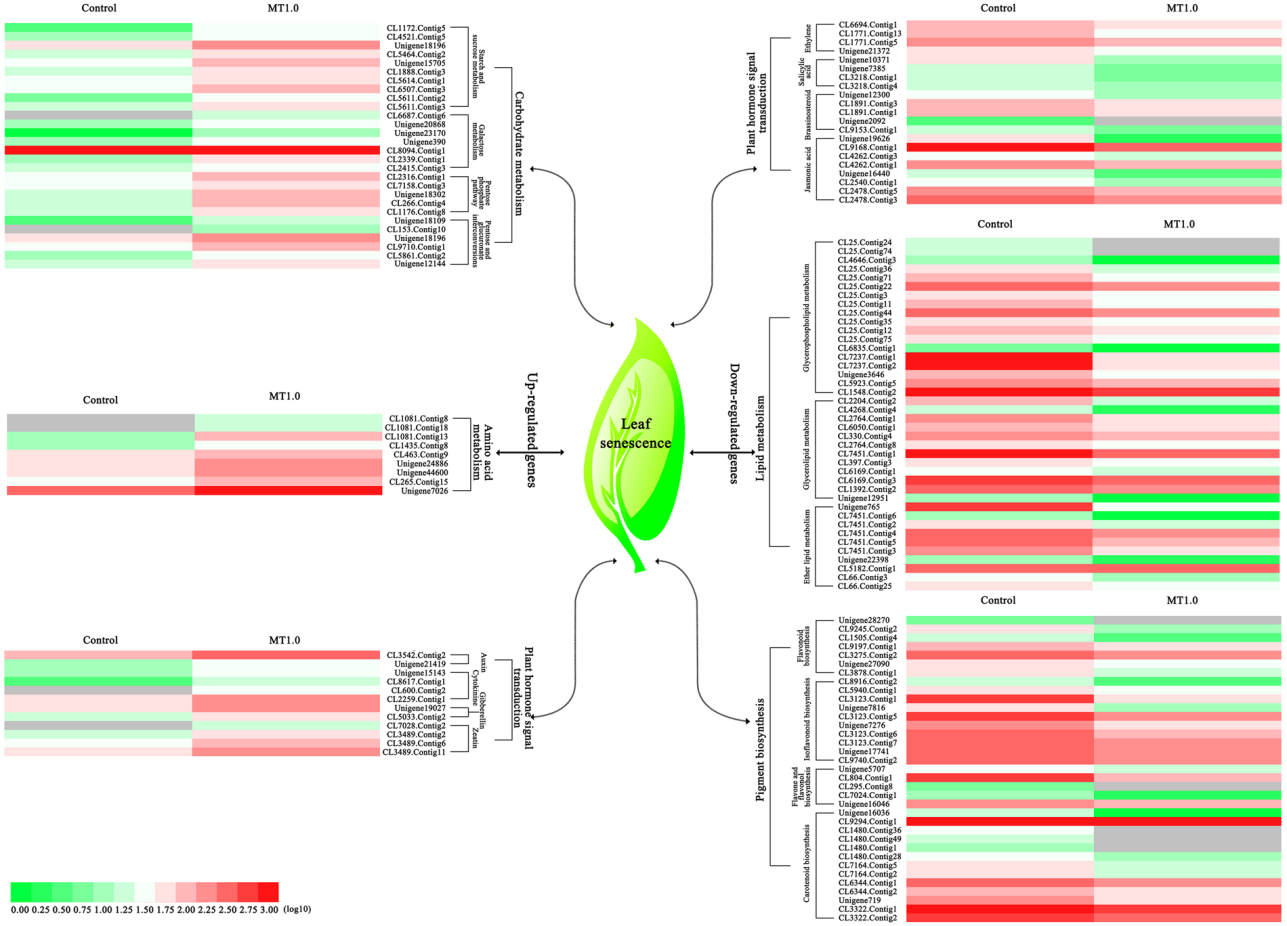
Heat map of main DEG expression patterns involved in carbohydrate metabolism, amino acid metabolism, lipid metabolism, plant hormone signal transduction and pigment biosynthesis. Annotation information of DEGs can be found in Table S4.

To elucidate the functions of these DEGs, they were assigned to GO categories and grouped into the following three main categories: biological process, cellular component and molecular function. Within the biological process category, 145 and 101 DEGs were enriched in metabolic process and cellular process. In the cellular component category, cell (74 DEGs) and cell part (74 DEGs) were dominant functions. In the molecular function category, catalytic activity (158 DEGs) and binding (98 DEGs) accounted for a major proportion (Fig. 11C). Subsequently, these DEGs were assigned in the KEGG pathway database, where 1,021 DEGs were mapped to 131 pathways and only 21 pathways met the condition of the Q-value ≤0.05 (Fig. 11D, Supplemental Table S3). Among them, metabolic pathways demonstrated the largest number with 489 DEGs (ko01100), followed by biosynthesis of secondary metabolites with 318 DEGs (ko01100) and starch and sucrose metabolism with 114 DEGs (ko00500). Ether lipid metabolism with 12 DEGs (ko00565) together with flavone and flavonol biosynthesis with 9 DEGs (ko00944) demonstrated the smallest number. On this basis, these pathways were partially divided into carbohydrate metabolism (starch and sucrose metabolism, galactose metabolism, pentose phosphate pathway, pentose and glucuronate interconversions), amino acid metabolism (cyanoamino acid metabolism), lipid metabolism (glycerophospholipid metabolism, glycerolipid metabolism, ether lipid metabolism), plant hormone signal transduction (plant hormone signal transduction, zeatin biosynthesis), pigment biosynthesis (flavonoid biosynthesis, isoflavonoid biosynthesis, flavone and flavonol biosynthesis, carotenoid biosynthesis), and others. The up-regulated DEGs were involved in carbohydrate metabolism, amino acid metabolism and plant hormone signal transduction (auxin, cytokinin, gibberellin and zeatin), whereas the down-regulated DEGs were contained in lipid metabolism, pigment biosynthesis and plant hormone signal transduction (ethylene, salicylic acid, brassinosteroid and jasmonic acid) (Fig. 12, Supplemental Table S4).

## Discussion

Leaf senescence is a highly regulated natural process during leaf development^69^, that can be accelerated by external factors, such as insufficient light, temperature change, pathogen attack, etc.^38^. As an extremely low light condition, darkness is an efficient method for inducing leaf senescence that can be effectively suppressed by exogenous melatonin treatment. Interestingly, different plant species showed specific responses in different concentrations of melatonin. For example, the addition of melatonin concentration between 20 and 100 µM to perennial ryegrass (*Lolium perenne* L.) was effective in suppressing leaf senescence^23^; barley (*Hordeum vulgare* L.) obtained an optimal response at 10 µM melatonin^33^ and 10 µM and 20 µM were the effective melatonin concentration for rice (*Oryza sativa* L.)^13^. Meanwhile, Wang *et al*. found that 10 mM melatonin was effective in delaying leaf senescence in apples^24^. In this study, the effects of different melatonin concentrations on leaf senescence were investigated, and the results showed increased *H* ^*o*^, SPAD values and Fv/Fm as well as lower MDA content, showing that melatonin clearly had a role in delaying dark-induced gardenia leaf senescence and that 1.0 mM concentration was the most effective one. These results also suggested that different plant species had different levels of sensitivity to melatonin, and the response in herbaceous plants was more intense than in woody plants, consistent with a previous study.

Leaf color turning from green to yellow is the most obvious symptom of leaf senescence^34^. In apples, exogenous melatonin treatment could slow leaf yellowing and keep the leaves green^24^. In our study, this same result was observed, and the MT1.0-treated gardenia leaves maintained their green color for a longer period. A previous study indicated that leaf senescence was largely due to a greater retention of carotenoids than green pigments^38^, and our results verified this conclusion. In gardenia leaves, dark-induced senescence was accompanied by a loss of chlorophyll content and an increase in carotenoid content; an increase in flavonoid content was also found, which suggested that gardenia leaf color turning from green to yellow during leaf senescence was mainly due to chlorophyll degradation and flavonoid and carotenoid synthesis. Furthermore, exogenous melatonin treatment could significantly maintain the chlorophyll and decrease the production of flavonoid and carotenoid in gardenia leaves; this result may be due to the scavenging of overproduced ROS was^23^. Additionally, the chlorophyll fluorescence parameters are the internal indication that plants have adapted to the ecological environment. Under high temperature stress, the declined F_v_/F_m_, F_v_/F_0_ and Y(II) were found in cucumber, but exogenous melatonin treatment could significantly suppress their declines^39^. Similar results were observed in this study, whereby decreases in F_v_/F_m_, F_v_/F_0_ and Y(II) in the control were more dramatic and each of these values were higher in MT1.0-treated leaves than in the control, which demonstrated that the exogenous melatonin treatment could effectively restrain the decline in potential efficiency of PSII of gardenia leaves exposed to darkness, alleviate damage to PSII, use absorbed light energy for photosynthetic electron transport to its maximum potential, and ultimately maintain the complete function of gardenia leaves^39^.

Leaf senescence induced by darkness was accompanied by excessive accumulation of ROS^40^. In this study, histochemical staining and fluorescence probes revealed rapid production of H_2_O_2_ and O_2_^−^ in gardenia leaves exposed to darkness. The excessive accumulation of ROS led to membrane lipid peroxidation, which could be assessed by REC and MDA content^41^. Here, the rapid accumulation of REC and MDA content was found in dark-induced gardenia leaves; moreover, the Pro content and GS activity also increased rapidly, whereas the opposite trend was observed for water and soluble protein contents. ROS is a mediator of programmed cell death (PCD) in plants^42^, and the PCD of gardenia leaves was also induced under darkness, manifested by the number of dead cells and cell dissolution. When exogenous melatonin was applied, the REC, MDA and Pro contents as well as PCD decreased, and water, solution protein contents and GS activity increased in dark-induced gardenia leaves. Moreover, exogenous melatonin treatment significantly decreased the accumulation of H_2_O_2_ and O_2_^−^. These results demonstrated that exogenous melatonin decreased cell membrane damage and PCD during dark treatment by reducing the accumulation of ROS. In addition, we also found that melatonin content was higher in all MT1.0-treated leaves compared with the control, indicating that exogenous melatonin treatment could effectively enhance the endogenous melatonin level; this result agreed with previous reports^23, 33, 43^, and it might be due to the exogenous melatonin treatment induced by the expression of melatonin biosynthesis genes. This hypothesis was supported by increased expression levels of *TDC* in gardenia leaves with exogenous melatonin treatment. As a substance with high antioxidant capacity, endogenous melatonin directly scavenged ROS^44, 45^. Moreover, the SOD-CAT pathway and AsA-GSH cycle are the main ROS scavenging systems in plants and can be activated by melatonin. Zhang *et al*. found a melatonin-activated SOD-CAT ROS scavenging pathway in perennial ryegrass^23^, and Wang *et al*. reported that melatonin scavenged ROS by regulating the AsA-GSH cycle in apples^24^. Our study indicated that exogenous melatonin significantly enhanced the SOD and CAT activities in gardenia leaves exposed to darkness, in agreement with the results of Zhang *et al*.^23^. However, in the AsA-GSH cycle, our results were not completely consistent with the work of Wang *et al*.^24^, and decreased ASA content, DHAR and MDHAR activities were found under exogenous melatonin treatment; this result might be due to increased APX activity scavenging more ROS by expending more AsA, an explanation given in previous work in strawberry plants (*Fragaria* × *ananassa* Duch.)^46^. Based on these results, the reduced cell membrane damage by exogenous melatonin was closely related to enhanced ROS scavenging by enhancing endogenous melatonin levels and regulating the SOD-CAT pathway and AsA-GSH cycle together.

The plant cuticle covering the leaf epidermis can prevent water loss into the atmosphere, and in grape, exogenous melatonin obviously enhances leaf cuticle thickness^26^. In the present study, there was no obvious difference in the length, width and opened degree of stomata between MT1.0-treated leaves and control, but the MT1.0-treated leaves had a more obvious corrugated texture in leaf cuticle, which suggested that exogenous melatonin treatment could prevent water loss from gardenia leaves. This speculation was confirmed by increased water content. Moreover, the mesophyll cell ultrastructures also changed under stress, and Zhao *et al*. found that the chloroplast appeared round and its membrane was blurred under high-temperature stress^47^. In this study, more intact mesophyll cell ultrastructures and more chloroplasts were observed in MT1.0-treated leaves, whereas the diffused membranes of some chloroplasts appeared in the control, in agreement with findings for grapes^26^. The validity of this result was supported by our observation that exogenous melatonin significantly restrained chlorophyll degradation and the decline of potential efficiency of photosystem II in gardenia leaves exposed to darkness.

Regarding the molecular regulatory mechanism of melatonin in plants, several in-depth studies have been undertaken using RNA-Seq.^11, 48–50^. In terms of melatonin delaying dark-induced leaf senescence, the expression patterns of chlorophyll degradation-related and senescence-associated genes were investigated in perennial ryegrass^23^ and apple^24^. Moreover, in addition to the relative transcript levels of chlorophyll degradation-related and senescence-induced genes in rice, Liang *et al*. also found using RNA-Seq that delayed leaf senescence was caused by altered expression levels of genes involved in oxidation–reduction, chlorophyll biosynthesis, pigment biosynthesis, stress responses, nutrient metabolism and remobilization^13^. In this study, to gain insight into the contribution of melatonin to leaf senescence, RNA-Seq was performed between the control and MT1.0-treated leaves with significantly different colors; 94,812 high-quality unigenes were obtained and 67,690 unigenes were annotated. Subsequently, 2,354 DEGs were identified including 782 up-regulated DEGs and 1,572 down-regulated DEGs. Q-PCR validated the RNA-Seq results as credible^11, 13, 48^. Among the DEGs, 1,021 DEGs were mapped to 131 pathways, and only 21 pathways met the condition of Q-value ≤0.05. These pathways were partially divided into six metabolisms including carbohydrate metabolism, amino acid metabolism, lipid metabolism, plant hormone signal transduction, pigment biosynthesis and others. In pigment biosynthesis, many down-regulated genes related to flavonoid biosynthesis and carotenoid biosynthesis were found, such as the flavonoid 3′,5′-hydroxylase gene (*F3*′,*5*′*H*), flavonol synthase gene (*FLS*), zeta-carotene desaturase gene (*ZDS*) and zeaxanthin epoxidase gene (*ZEP*), which suggested that flavonoid and carotenoid biosynthesis were inhibited by exogenous melatonin; this result was validated by our observation that exogenous melatonin significantly inhibited an increase of flavonoid and carotenoid contents. Plant survival was closely related to carbohydrates, which are the substrate for respiration^51^. In this study, many up-regulated genes related to carbohydrate metabolism were obtained, such as beta-glucosidase gene (*GLU*), starch synthase gene (*SS*), beta-galactosidase gene (*GAL*), phosphoglucomutase gene (*PGM*) and stachyose synthetase gene (*STS*). In cassava (*Manihot esculenta* Crantz.), many starch synthesis-related genes were inhibited during the postharvest physiological deterioration process, and melatonin resulted in the down-regulation of these genes^49^. Our results were contrary to this conclusion, which might be due to melatonin-mediated carbohydrate metabolism providing more energy for various biological processes^52^. Moreover, amino acid metabolism and lipid metabolism were closely related to leaf senescence^38^. In this study, exogenous melatonin resulted in the up-regulation of genes in amino acid metabolism and the down-regulation of genes in lipid metabolism in gardenia leaves exposed to darkness; the former agreed with increased solution protein content. Additionally, hormones are an important signalling substance in plants that can be induced by plant development signals and environmental factors to regulate leaf senescence^32^. In response to exogenous melatonin, auxin, cytokinin, gibberellin, zeatin, ethylene, salicylic acid, brassinosteroid and jasmonic acid involved in plant hormone signal transduction could be induced in gardenia leaves, because many DEGs involved in these hormone biosynthetic pathways were found. These hormones had different roles in regulating leaf senescence. Noh and Amasino found that spraying exogenous auxin could inhibit the expression of senescence-associated genes^53^. Gan and Amasino found that cytokinin content gradually decreased during leaf senescence^54^, exogenous spraying experiment and genetically modified studies had all proved that cytokinin delay of leaf senescenc^55^, and that senescence was impeded by exogenous gibberellic acid^55^. These findings demonstrated that auxin, cytokinin and gibberellin could inhibit leaf senescence. Similarly, as a natural cytokinin, zeatin could maintain leaf function and inhibit leaf senescence^56^. Moreover, ethylene, salicylic acid, brassinosteroid and jasmonic acid could promote leaf senescence^57–60^. In this study, DEGs involved in auxin, cytokinin, gibberellin and zeatin were up-regulated, down-regulated DEGs were found in ethylene, salicylic acid, brassinosteroid and jasmonic acid under exogenous melatonin treatment. These results demonstrated that exogenous melatonin treatment could significantly altered the expression levels of numerous genes involved in plant hormone signal transduction, which could altered the synthesis of these corresponding hormones to delay dark-induced leaf senescence.

**Figure 13:**
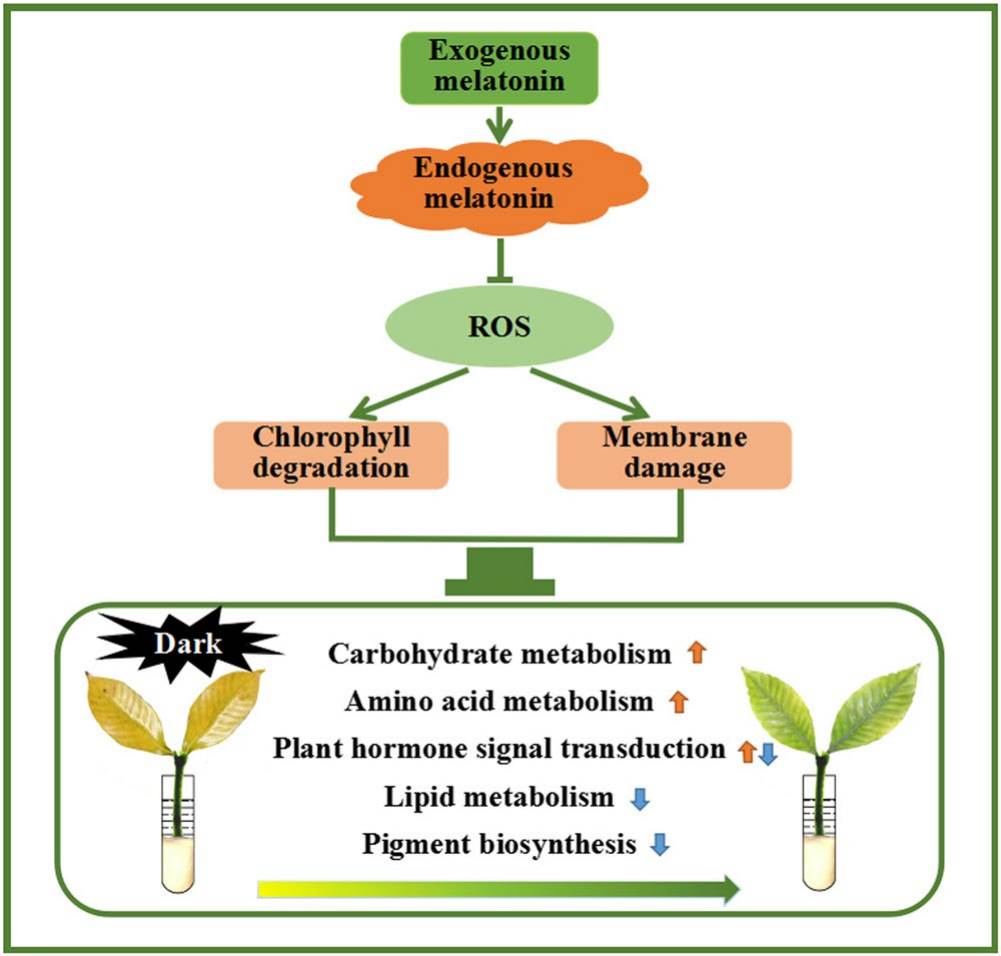
Proposed pathways of dark-induced leaf senescence in response to exogenous melatonin application in gardenia.

In summary, exogenous melatonin treatment could significantly delay the dark-induced senescence of gardenia leaves, and 1.0 mM concentration had the best effect. As depicted in Fig. 13, the treatment of exogenous melatonin effectively enhanced the endogenous melatonin content by increasing the expression levels of *TDC*. Melatonin was an antioxidant that could regulate the SOD-CAT pathway and AsA-GSH cycle together to scavenge the overproduction of ROS. Subsequently, chlorophyll degradation and membrane damage were reduced as manifested by lower chlorophyll, REC and MDA contents and PCD compared with the control. Additionally, the expression levels of numerous genes involved in carbohydrate metabolism, amino acid metabolism, lipid metabolism, plant hormone signal transduction, pigment biosynthesis and others were significantly altered in response to delayed dark-induced gardenia leaf senescence via exogenous melatonin treatment. These results provide a new opportunity for the use of melatonin in improving the ornamental value of cut gardenia foliage.

## Materials and Methods

### Plant materials and treatments

Gardenia (*Gardenia jasminoides* Ellis) was grown in the Wenhui street campus of Yangzhou University, Jiangsu Province, China (32°39′ N, 119°43′ E). A 4.0-cm stem with two fully mature leaves was collected as the treated material and taken to our laboratory timely. After washing the samples with tap water and sterile double-distilled water for 5 min, they were dried with blotting paper. To prevent gas bolt, the lower part of stems were cut 1.0 cm in length underwater. Subsequently, the stems were wrapped with the absorbent cotton and then inserted into 5.0-mL centrifuge tubes containing either sterile double-distilled water or a melatonin solution (0.05, 0.1, 1.0 and 2.0 mM) (Sigma-adlrich, USA). They were transferred together to a dark room with 25 °C and 60% relative humidity, supplied with 4 mL sterile double-distilled water or a melatonin solution and the liquid was replaced every day at a fixed time. After an 18-day treatment, *H°*, SPAD value, chlorophyll fluorescence parameters and MDA content were determined to identify the optimal concentration. Based on this information, the same treatment was performed with MT1.0, and the leaves were sampled separately on 0, 8, 16 and 24 days after treatment. Among these samples, MT1.0-treated leaves and control significantly different colors on 24 days were used for the RNA-Seq analysis separately, and the other leaves were used for other indices measurements. After determination of chlorophyll fluorescence parameters, *H°*, SPAD and PCD, one part was fixed in 3% glutaraldehyde for anatomy observations, and the others were immediately frozen in liquid nitrogen, and then stored at −80 °C until further analysis.

### Color indices measurement

The color indices of leaves were measured with a hand-held RM200QC spectrocolorimeter (X-Rite, Switzerland) using two color parameters including a* and b* values. The hue angle (*H°* = arctangent (b*/a*)) were calculated. The SPAD values of leaves were measured by a chlorophyll meter (SPAD-502, Japan).

### Melatonin content measurement

Before melatonin content was detected, the samples were pretreated. Firstly, 0.1 g leaf was ground to a fine powder with liquid nitrogen and extracted with 1.0 mM phosphate buffer (pH 7.2 and containing 5% methanol) in 1.5 mL centrifuge tube. Secondly, the mixed sample was moved on ice and undergone ultrasound at 100 W for 30 s using an ultrasonic instrument (VCX-130, Sonics, USA), and then the extract was centrifuged at 4 °C for 10 min at 10,000 × g and the resulting supernatant. Subsequently, the melatonin was detected according to the guideline of an ELISA kit (Shanghai Qiaodu Biotechnology Co., Ltd., China) and its content was obtained by the SpectraMax M5 plate reader (Molecular Devices Corporation, USA).

### Pigment contents and chlorophyll fluorescence parameters measurement

Chlorophyll and carotenoid contents were all determined according to the methods reported by Zou^61^. 0.1 g leaves were collected and placed in the test tubes, then added 15 mL 80% (v/v) acetone and placed for one night. Chlorophyll and carotenoid concentrations were calculated from spectroscopy absorbnce measurements at 663.2, 646.8, and 470 nm. Moreover, flavonoid content was measured by a reagent kit (Suzhou Comin Biotechnology Co., Ltd., China). Additionally, the chlorophyll fluorescence parameters were measured with a chlorophyll fluorescence spectrometer (Heinz Walz GmbH 91090 Effeltrich, Germany). In the measurement, the leaves were first placed in the dark for 30 minutes,Then turn on the measurement light (0.5 μmol m ^−2^ s ^−1^) and record the minimum fluorescence (Fo) after dark adaptation. Next, a saturation pulse (8000 μmol m ^−2^ s ^−1^) with a duration of only 0.2–1.5 s was opened, measuring the maximum fluorescence (Fm) after dark adaptation.According to the measured parameters, calculating the potential quantum yield of PSII (Fv/Fo = (Fm-Fo)/Fo) and maximum quantum yield of PSII (Fv/Fm = (Fm-Fo)/Fm)^62^. Then, the slow fluorescence induced kinetic curve was measured. The system automatically turned on the actinic light and the measured light. The real-time fluorescence value F was measured before the saturation pulse, and 1 time saturation pulse was irradiated every 20 s to measure Fm′.According to Fm′ and F, the actual photosynthetic efficiency of photosystem II could be taken. (Y (II) = ΔF/Fm′ = (Fm′-F)/Fm′)^63^.

### Physiological indices measurement

H_2_O_2_ accumulation was detected by DAB staining^64^. Briefly, the leaves were immersed in 0.1 mg/mL DAB in 50 mM Tris-acetate buffer, pH 5.0, at 25 °C for 24 h in the dark, and then they were boiled in 95% (v/v) ethanol for 15 min and photographed using a camera (Canon 50D, Japan).

O_2_^−^ accumulation was detected by a reagent kit (Shanghai Haling Biotechnology Co., Ltd., China). The samples were observed at 540 nm excitation wavelength and 590 nm emanation wavelength, and imaged with a fluorescent microscope (Axio Imager D2, ZEISS, Germany). The fluorescent signals were gathered using ZEN software (ZEISS, Germany).

Water content and REC were determined according to the method reported by Yang *et al*.^65^. Fresh samples were washed with deionized water and perforated using a 1 cm diameter punch. 0.1 g leave was placed in a test tube and 20 mL of deionized water was added. After standing for 4 h at room temperature, the initial solution conductivity C1 was measured. Then, the tube was heated in boiling water for 30 min, and the conductivity C2 was measured after shaking. Relative Conductivity = C1 / C2 × 100%. MDA, soluble protein and Pro contents were evaluated according to the guidelines of reagent kits (Nanjing Jiancheng Bioengineering Institute, China), and glutamate synthetase (GS, EC 6.3.1.2) activity was evaluated by a reagent kit (Suzhou Comin Biotechnology Co., Ltd., China). We use unit “U” to indicate the enzyme activity. The amount of enzymatic conversion of 1μmol substrate in 1 min is called 1 enzyme unit (U).

### PCD detection

Leaf senescence was a progress of PCD^32^, and we firstly used the Evans blue staining solution to stain the dying cells without intact cell membranes blue^37^. The samples were stained with 0.1% Evans blue staining solution (Shanghai Yuanye biological technology Co., Ltd., China) for 2 min and then washed with double-distilled water. After drying with blotting paper, they were observed by a light microscope (Olympus CX31RTSF, Tokyo, Japan). Secondly, the isolated mesophyll cells were observed. The 1–2mm excised leaves were put in the sterile flask, and 3 mL enzyme solution (2% cellulose, 0.6% macerozyme, 0.6 mol/L mannitol, 5.0 mmol/L CaCl_2_, 1.0 mmol/L KH_2_PO_4_, 4 g/L PVP, pH5.8) was added. After sealing, they were stored at 28 °C for 6 h under darkness. Subsequently, they were filtered using a 45 μm mesh screen, and the liquid mixture was transferred to a sterile centrifuge tube and centrifuged at 1,000 r/min. The precipitate was washed with 0.14 mol/L mannitol (pH5.8) for 2–3 times, and then dissolved in mannitol. The resulting suspension containing mesophyll cells was observed by a light microscope (Olympus CX31RTSF, Tokyo, Japan).

### Antioxidant enzyme activities and AsA–GSH cycle measurement

When antioxidant enzyme activities were measured, firstly, 0.5 g leaf was ground to a fine powder with liquid nitrogen and extracted with ice-cold 50 mM phosphate buffer (pH 7.8). The extracts were centrifuged at 4 °C for 15 min at 10,000 × g and then the resulting supernatants; thereafter referred to as crude extracts, was collected and used for enzyme activities assay^61^. SOD and CAT activities were evaluated by reagent kits (Nanjing Jiancheng Bioengineering Institute, China).

Antioxidant and key enzymes that involved in AsA-GSH cycle contained ASA, GR, DHAR, MDHAR and APX. Firstly, 0.1 g leaf was ground to 10% tissue homogenate with reagent of kits or 0.9% saline, and the supernatant could be obtained by centrifugation. Moreover, ASA content, GR, DHAR and MDHAR activities were evaluated by reagent kits (Suzhou Comin Biotechnology Co., Ltd., China), and APX activity was also evaluated by a reagent kit (Nanjing Jiancheng Bioengineering Institute, China).

### Anatomy observation

The surfaces of leaves were observed by the environmental scanning electron microscopy (Philips XL-30 ESEM, Holland). The fixed leaves were firstly dehydrated in a gradient ethanol solution and treated with the mixtures of acetone: anhydrous alcohol (1:1, 2:1, 1:0, v/v), acetone: isoamyl acetate (1:1, 1:2, v/v), and pure isoamyl acetate. After drying and spraying gold (EIKO IB-3, Hitachi, Japan), the samples were observed.

Moreover, the anatomical details of leaves were observed by the transmission electron microscope (Tecnai 12, Philips, Holland). The fixed leaves were washed 3 times with 0.1 mol/L phosphate buffer for 15 min, and post-fixed with 1% osmium tetroxide for 4 h at room temperature (25 °C). After washing 3 times with 0.1 mol/L phosphate buffer for 15 min each, the leaves were dehydrated using 50%, 70%, 85%, 95% and 100% gradient ethanol for 15 min each. Moreover, they were treated with 100% acetone solution (15 min) and acetone solution containing anhydrous sodium sulfate (15 min), infiltrated in Spurr resin and then hardened at 70 °C for 24 h. Sections (70 nm thick) were cut with a diamond knife using a Leica EM UC6 ultramicrotome (Leica Co., Austria) and stained with 1% uranyl acetate in 70% methanol, and 1% lead citrate before examination. Finally, the samples were observed and imaged.

### RNA-Seq and data analysis

Total RNAs extracted from the leaves of the control and 24-day MT1.0 treatment under darkness using a MiniBEST Plant RNA Extraction Kit (TaKaRa, Japan) were used for transcriptome sequencing. The concentration and purity of the total RNA were calculated using a spectrophotometer (Eppendorf, Germany) with absorbance of 260 and 280 nm. Six libraries (Control and MT1.0, three replicates) were prepared and sequenced by Beijing Genomic Institute (Shenzhen, China) using an Illumina HiSeq™ 2000 platform (Illumina Inc., San Diego, CA, USA). After raw reads filtering, transcriptome *de novo* assembly was performed using the short reads assembling program Trinity, which combined three independent software modules: Inchworm, Chrysalis and Butterfly^66^. The resulting sequences of Trinity were called Unigenes. Unigene annotation was performed using various bioinformatics databases, including NR, NT, GO, COG, KEGG, Swiss-Prot and Interpro.

The unigene expression level was calculated using the fragments per kilo bases per million reads (FPKM) method^67^. DEGs were defined based on fold change ≥2.0 and adjusted P-value ≤0.05. The confirmed DEGs were subjected to GO functional analysis and KEGG pathway analysis.

### Gene expression analysis

Gene transcript levels were analyzed using Q-PCR with a BIO-RAD CFX Connect^TM^ Optics Module (Bio-Rad, USA). The cDNA was synthesized from RNA using PrimeScript^®^ RT reagent Kit With gDNA Eraser (TaKaRa, Japan). *Actin* was used as an internal control in gardenia (forward primer: 5′-GCGAGGAAACAAGTGGAAGACTA-3′; reverse primer: 5′-TGCCAACCACCATTTATTAGGAG-3′)^29^. All gene-specific primers in this study were shown in Supplemental Table S1, and synthesized by Shanghai Sangon Biological Engineering Technology & Services Co., Ltd. (Shanghai, China). Q-PCR was performed using the SYBR^®^ Premix Ex Taq^TM^ (Perfect Real Time) (TaKaRa, Japan) and contained 12.5 µL 2 × SYBR Premix Ex Taq^TM^, 2 µL cDNA solution, 2 µL mix solution of target gene primers and 8.5 µL ddH_2_O in a final volume of 25 µL. The amplification was carried out under the following conditions: 50 °C for 2 min followed by an initial denaturation step at 95 °C for 30 s, 40 cycles at 95 °C for 5 s, 50 °C for 15 s, and 72 °C for 30 s. Gene relative expression levels of target genes were calculated by the 2^−△△Ct^ comparative threshold cycle (Ct) method^68^. The Ct values of the triplicate reactions were gathered using the Bio-Rad CFX Manager V1.6.541.1028 software.

### Statistical analysis

All experiments described here were repeated three times arranged in a completely randomized design. Sequences analysis was performed by DNAMAN 5.0 software. Primers were designed using a Primer 5.0 program. All data were means of three replicates with standard deviations. The results were analyzed for variance using the SAS/STAT statistical analysis package (version 6.12, SAS Institute, Cary, NC, USA).

## Electronic supplementary material

Supplementary 1

## References

[1] Lerner AB, Case J, Takahashi Y, Lee TH, Mori W (1958). Isolation of melatonin, the pineal gland factor that lightens melanocytes. J Am Chem Soc..

[2] Hattori A (1995). Identification of melatonin in plants and its effects on plasma melatonin levels and binding to melatonin receptors in vertebrates. Biochem Mol Biol Int..

[3] Dubbels R (1995). Melatonin in edible plants identified by radioimmunoassay and by high performance liquid chromatographymass spectrometry. J Pineal Res..

[4] Chen G (2003). Melatonin in Chinese medicinal herbs. Life Sci..

[5] Tan DX (2012). Functional roles of melatonin in plants, and perspectives in nutritional and agricultural science. J Exp Bot..

[6] Tan DX, Manchester LC, Esteban-Zubero E, Zhou Z, Reiter RJ (2015). Melatonin as a potent and inducible endogenous antioxidant: synthesis and metabolism. Molecules.

[7] Byeon Y (2015). Coordinated regulation of melatonin synthesis and degradation genes in rice leaves in response to cadmium treatment. J Pineal Res..

[8] Rajniak J, Barco B, Clay NK, Sattely ES (2015). A new cyanogenic metabolite in *Arabidopsis* required for inducible pathogen defence. Nature.

[9] Zhang N (2013). Melatonin promotes water-stress tolerance, lateral root formation, and seed germination in cucumber (*Cucumis sativus* L.). J Pineal Res..

[10] Pelagio-Flores R, Muñoz-Parra E, Ortiz-Castro R, López-Bucio J (2012). Melatonin regulates *Arabidopsis* root system architecture likely acting independently of auxin signaling. J Pineal Res..

[11] Zhang N (2014). The RNA-seq approach to discriminate gene expression profiles in response to melatonin on cucumber lateral root formation. J Pineal Res..

[12] Kolář J, Johnson CH, Macháčková I (2003). Exogenously applied melatonin (*N*-acetyl-5-methoxytryptamine) affects flowering of the short-day plant *Chenopodium rubrum*. Physiol Plant.

[13] Liang C (2015). Melatonin delays leaf senescence and enhances salt stress tolerance in rice. J. Pineal Res..

[14] Bajwa VS, Shukla MR, Sherif SM, Murch SJ, Saxena PK (2014). Role of melatonin in alleviating cold stress in *Arabidopsis thaliana*. J Pineal Res..

[15] Xu XD, Sun Y, Sun B (2010). Effects of exogenous melatonin on active oxygen metabolism of cucumber seedlings under high temperature stress. Chin J Appl Ecol..

[16] Li C (2012). The mitigation effects of exogenous melatonin on salinity-induced stress in *Malus hupehensis*. J Pineal Res..

[17] Posmyk MM, Kuran H, Marciniak K, Janas KM (2008). Presowing seed treatment with melatonin protects red cabbage seedlings against toxic copper ion concentrations. J Pineal Res..

[18] Liu N (2015). Sodic alkaline stress mitigation with exogenous melatonin involves reactive oxygen metabolism and ion homeostasis in tomato. Sci Hortic..

[19] Li MQ (2016). Melatonin mediates selenium-induced tolerance to cadmium stress in tomato plants. J Pineal Res..

[20] Afreen F, Zobayed SMA, Kozai T (2006). Melatonin in Glycyrrhiza uralensis: response of plant roots to spectral quality of light and UV-B radiation. J Pineal Res..

[21] Yin L (2013). Exogenous melatonin improves *Malus* resistance to Marssonina apple blotch. J. Pineal Res..

[22] Zhao H (2015). Melatonin regulates carbohydrate metabolism and defenses against *Pseudomonas syringae* pv. *tomato DC3000* infection in *Arabidopsis thaliana*. J. Pineal Res..

[23] Zhang J, Li H, Xu B, Li J, Huang BR (2016). Exogenous melatonin suppresses dark-induced leaf senescence by activating the superoxide dismutase-catalase antioxidant pathway and down-regulating chlorophyll degradation in excised leaves of perennial ryegrass (*Lolium perenne* L.). Front Plant Sci..

[24] Wang P (2012). Delayed senescence of apple leaves by exogenous melatonin treatment: toward regulating the ascorbate-glutathione cycle. J Pineal Res..

[25] Lei Q (2013). Identification of genes for melatonin synthetic enzymes in ‘Red Fuji’ apple (*Malus domestica Borkh*. *cv*. *Red*) and their expression and melatonin production during fruit development. J Pineal Res..

[26] Meng JF (2014). The ameliorative effects of exogenous melatonin on grape cuttings under water-deficient stress: antioxidant metabolites, leaf anatomy, and chloroplast morphology. J Pineal Res..

[27] Zhao Y (2012). Melatonin and its potential biological functions in the fruits of sweet cherry. J Pineal Res..

[28] Han JP, Chen SL, Zhang WS, Wang YY, Li XY (2007). Study on genetic diversity and differentiation of *gardenia jasminoides* Ellis using RAPD markers. *Chin*. Pharm J..

[29] Tsanakas GF, Manioudaki ME, Economou AS, Kalaitzis P (2014). De novo transcriptome analysis of petal senescence in *Gardenia jasminoides* Ellis. BMC Genomics.

[30] Causin HF (2009). Changes in hydrogen peroxide homeostasis and cytokinin levels contribute to the regulation of shade-induced senescence in wheat leaves. Plant Sci..

[31] Brouwer B, Ziolkowska A, Bagard M, Keech O, GARDESTRÖM P (2012). The impact of light intensity on shade-induced leaf senescence. Plant Cell Environ..

[32] Chen, X. Y. & Xue, W. H. Plant physiology and molecular biology(ed. Guo F. Q.) Plant leaf senescence and its regulation. 514-522 Higher Education Press, Beijing, Chinese (2012).

[33] Arnao M, Hernández-Ruiz J (2009). Protective effect of melatonin against chlorophyll degradation during the senescence of barley leaves. J Pineal Res..

[34] Ke X (2015). Exogenous application of melatonin delays leaf senescence in adzuki bean. J Heilongjiang Bayi Agric Univ..

[35] Wang Z, Gerstein M, Snyder M (2009). RNA-Seq: a revolutionary tool for transcriptomics. Nat Rev Genet..

[36] Yang CX, Zhang T, Xu M, Zhu PL, Deng SY (2016). Insights into biosynthetic genes involved in the secondary metabolism of *Gardenia jasminoides* Ellis using transcriptome sequencing. Biochem Syst Ecol..

[37] Young T, Gallie D (1999). Analysis of programmed cell death in wheat endosperm reveals differences in endosperm development between cereals. Plant Mol Biol..

[38] Smart CM (1994). Gene expression during leaf senescence. New Phytol..

[39] Xu, X., Sun, Y., Guo, X., Sun, B. & Zhang, J. Effect of exogenous melatonin on photosynthesis and chlorophyll fluorescence parameters in leaves of cucumber seedlings under high temperature stress. *J Nucl Agric Sci*. **25**(1), 179–184 (2011).

[40] Buchanan-Wollaston V (2003). The molecular analysis of leaf senescence - a genomics approach. Plant Biotechnol J..

[41] Bhattacharjee S (2014). Membrane lipid peroxidation and its conflict of interest: the two faces of oxidative stress. Curr Sci..

[42] Jabs T (1999). Reactive oxygen intermediates as mediators of programmed cell death in plants and animals. Biochem Pharmacol..

[43] Fan J (2015). Alleviation of cold damage to photosystem II and metabolisms by melatonin in Bermudagrass. Front Plant Sci..

[44] Pieri C, Marra M, Moroni F, Recchioni R, Marcheselli F (1994). Melatonin: a preoxyl radical scavenger more effective than vitamin E. Life Sci..

[45] Pieri C, Moroni F, Marra M, Marcheselli F, Recchioni R (1995). Melatonin is an efficient antioxidant. Arch Gerontol Geriatr..

[46] Luo Y, Tang HR, Zhang Y (2007). Effect of low temperature stress on activities of SOD and enzymes of ascorbate-glutathione cycle. Acta Hortic Sin..

[47] Zhao D, Han C, Zhou C, Tao J (2015). Shade ameliorates high temperature-induced inhibition of growth in herbaceous peony (*Paeonia lactiflora* Pall). Int J Agric Biology..

[48] Weeda S (2014). Arabidopsis transcriptome analysis reveals key roles of melatonin in plant defense systems. PLoS ONE.

[49] Hu W (2016). Comparative physiological and transcriptomic analyses reveal the actions of melatonin in the delay of postharvest physiological deterioration of cassava. Front Plant Sci..

[50] Yuan S (2016). RNA-seq analysis of overexpressing ovine *AANAT* gene of melatonin biosynthesis in switchgrass. Front Plant Sci..

[51] Shi MF (2010). A review of the correlation of flooding adaptability and carbohydrates in plants. Chin J Plant Ecol..

[52] Li MH (2008). Nitrogen and carbon source-sink relationships in trees at the Himalayan tree lines compared with lower elevations. Plant cell environ..

[53] Noh YS, Amasino RM (1999). Identification of a promoter region responsible for the senescence-specific expression of *SAG12*. Plant Mol Biol..

[54] Gan SS, Amasino RM (1995). Inhibition of leaf senescence by autoregulated production of cytokinin. Science.

[55] Yu K, Wei J, Ma Q, Yu D, Li J (2009). Senescence of aerial parts is impeded by exogenous gibberellic acid in herbaceous perennial *Paris polyphylla*. J Plant Physiol..

[56] Zhao C, Kang S, Wang J, Guo X, Li H (2000). Research on phyto-hormones regulating mechanism of the senescence of wheat leaves. Acta Agric Boreali -Sinica..

[57] Abeles FB (1988). Induction of 33-kD and 60-kD peroxidases during ethylene-induced senescence of cucumber cotyledons. Plant Physiol..

[58] Besseau S, Li J, Palva ET (2012). WRKY54 and WRKY70 cooperate as negative regulators of leaf senescence in *Arabidopsis thaliana*. J Exp Bot..

[59] Jibran R, Hunter DA, Dijkwel PP (2013). Hormonal regulation of leaf senescence through integration of developmental and stress signals. Plant Mol Biol..

[60] Xiao, S., et al. COS1: an *Arabidopsis coronatine insensitive1* suppressor essential for regulation of jasmonate-mediated plant defense and senescence. *Plant Cell* **16**, 1132-1142 (2004). 10.1105/tpc.020370 PMC423205 15075400

[61] Zou, Q. Plant Physiology Experimental Guidance. China Agricultural Press, Beijing, Chinese (2000).

[62] Kooten OV, Snel JFH (1990). The use of chlorophyll fluorescence nomenclature in plant stress physiology. Photosynth Res..

[63] Genty B, Briantais JM, Baker NR (1989). The relationship between the quantum yield of photosynthetic electron transport and quenching of chlorophyll fluorescence. Biochimica et Biophysica Acta..

[64] Tian, F., et al. Enhanced stability of thylakoid membrane proteins and antioxidant competence contribute to drought stress resistance in the tasg1 wheat stay-green mutant. *J Exp Bot*. **64**, 1509–1520 (2013). 10.1093/jxb/ert004 PMC3617820 23378376

[65] Yang GP, Rhodes D, Joly RJ (1996). Effects of high temperature on membrane stability and chlorophyll fluorescence in glycinebetaine-deficient and glycinebetaine-containing maize lines. Funct Plant Biol..

[66] Grabherr MG (2011). Full-length transcriptome assembly from RNA-Seq data without a reference genome. Nat Biotechnol..

[67] Mortazavi A, Williams BA, McCue K, Schaeffer L, Wold B (2008). Lorian schaeffer and barbara wold mapping and quantifying mammalian transcriptomes by RNA-Seq. Nat Methods.

[68] Schmittgen TD, Livak KJ (2008). Analyzing real-time PCR data by the comparative CT method. Nat Protoc..

[69] Lim PO, Kim HJ, Nam HG (2007). Leaf senescence. Annu Rev Plant Biol..

