# Supplementary material for: Ameliorative effects of melatonin on dark-induced leaf senescence in gardenia (Gardenia jasminoides Ellis): leaf morphology, anatomy, physiology and transcriptome
Source: Sci Rep. 2017 Sep 5;7:10423. doi: 10.1038/s41598-017-10799-9 (PMC5585368; doi:10.1038/s41598-017-10799-9)
Supplement: Supplementary file 1 — Supplementary 1 [file 41598_2017_10799_MOESM1_ESM.doc]

Ameliorative effects of melatonin on dark-induced leaf senescence in gardenia (*Gardenia jasminoides* Ellis): leaf morphology, anatomy, physiology and transcriptome

Daqiu Zhao, Rong Wang, Jiasong Meng, Zhiyuan Li, Yanqing Wu, and Jun Tao*

Supplemental Table S1 Primers used for Q-PCR.

| Gene ID | Forward primer (5' - 3') | Reverse primer (5' - 3') |
| --- | --- | --- |
| CL712.Contig1 | GTCACCGTTTCTTCTCCC | TAAAGTTCATCCGCTCCC |
| CL3322.Contig1 | ATACGAGCGGGAGGAGAT | CCTTGTTAATGAGCACGAAA |
| CL4262.Contig1 | TGAATCCTGCTATTTCTGAG | GTGCTTTACCCTTACCGA |
| CL5375.Contig1 | GCCAGAATCATAGACCCTT | CAGCCACGAGAAAACCAT |
| CL7451.Contig6 | GTCAGATTCTTCACCTCCC | ACAAGCCGCTCATATACATA |
| CL7992.Contig1 | GACAGACGATAAAGAAGCATAC | AGAGACATAAGAGCAGGTGG |
| Unigene4123 | CTGTGATGTGTGGAGGCT | TTGTTCTGTTCTGGCTGTC |
| Unigene5101 | TAGGGATAGGCAAGTTAGAGT | AGAGGAGGGTCAACAAGAT |
| Unigene5138 | TACACGACTGACTAATGGGA | GATAGAGAGAAGATGTGAATGC |
| Unigene7142 | AAAGAGGGGTTAGGTAGGG | GATTTTGAAGGACGGAAGA |
| Unigene10058 | CTCAAAACTCCACCATCG | TCTGCTTCGCTATCACTTC |
| Unigene11096 | GAATCAGGAGGAACAAAGC | TCGCCAGTATGACCAGTG |
| Unigene12872 | CTGTTCATCCATCGGTTA | TTGTTTGGTGTCCCTTAC |
| Unigene24886 | GGCGTTAGAGCCTGATTG | TAGTTGGTTGCGTTGTCG |
| Tryptophan decarboxylase gene | CTGGGAGAAATGTTATGC | ATGAAGGACTTTGGGAGC |
| Actin | GCGAGGAAACAAGTGGAAGACTA | TGCCAACCACCATTTATTAGGAG |

**Supplemental Table S2 Effect of MT1.0 on fluorence intensity value** **of O_2_^·-^ accumulation during dark-induced gardenia leaf senescence**.

| Treated time (days) | Fluorence intensity value (a.u.) | |
| --- | --- | --- |
|  | Control | MT1.0 |
| 0 | 11.28±0.63f | 11.37±0.44f |
| 8 | 19.06±0.24de | 17.52±0.67e |
| 16 | 37.89±0.33b | 20.48±1.50d |
| 24 | 47.01±0.50a | 28.52±0.47c |

The values represented the mean ± SD, and different letters indicate significant differences according to Duncan’s multiple range test (*P* < 0.05).

**Supplemental Table S3 KEGG enrichment of the annotated DEGs**.

| No. | Pathway | DEGs with pathway annotation | Q-value | Pathway ID |
| --- | --- | --- | --- | --- |
| 1 | Phenylpropanoid biosynthesis | 96 (5.84%) | 6.650920E-24 | ko00940 |
| 2 | [Starch and sucrose metabolism](../../../../F:%5C%E7%A7%91%E7%A0%94%5C%E5%AE%9E%E9%AA%8C%5C2016%5C%E8%A4%AA%E9%BB%91%E7%B4%A0%5C%E6%A0%80%E5%AD%90%5C%E8%BD%AC%E5%BD%95%E7%BB%84%5CTranscriptome_Denovo_Report%5CTranscriptome_Denovo_Report%5CBGI_result%5C5.Quantify%5CDifferentExpressedGene%5CFunctional_Enrichment%5CPathway%5CControl-VS-MT10.DEseq2_Method.htm#gene2%23gene2) | 114 (6.94%) | 1.153063E-23 | ko00500 |
| 3 | [Cyanoamino acid metabolism](../../../../F:%5C%E7%A7%91%E7%A0%94%5C%E5%AE%9E%E9%AA%8C%5C2016%5C%E8%A4%AA%E9%BB%91%E7%B4%A0%5C%E6%A0%80%E5%AD%90%5C%E8%BD%AC%E5%BD%95%E7%BB%84%5CTranscriptome_Denovo_Report%5CTranscriptome_Denovo_Report%5CBGI_result%5C5.Quantify%5CDifferentExpressedGene%5CFunctional_Enrichment%5CPathway%5CControl-VS-MT10.DEseq2_Method.htm#gene3%23gene3) | 57 (3.47%) | 1.074703E-17 | ko00460 |
| 4 | [Biosynthesis of secondary metabolites](../../../../F:%5C%E7%A7%91%E7%A0%94%5C%E5%AE%9E%E9%AA%8C%5C2016%5C%E8%A4%AA%E9%BB%91%E7%B4%A0%5C%E6%A0%80%E5%AD%90%5C%E8%BD%AC%E5%BD%95%E7%BB%84%5CTranscriptome_Denovo_Report%5CTranscriptome_Denovo_Report%5CBGI_result%5C5.Quantify%5CDifferentExpressedGene%5CFunctional_Enrichment%5CPathway%5CControl-VS-MT10.DEseq2_Method.htm#gene4%23gene4) | 318 (19.35%) | 4.924352E-14 | ko01110 |
| 5 | [Metabolic pathways](../../../../F:%5C%E7%A7%91%E7%A0%94%5C%E5%AE%9E%E9%AA%8C%5C2016%5C%E8%A4%AA%E9%BB%91%E7%B4%A0%5C%E6%A0%80%E5%AD%90%5C%E8%BD%AC%E5%BD%95%E7%BB%84%5CTranscriptome_Denovo_Report%5CTranscriptome_Denovo_Report%5CBGI_result%5C5.Quantify%5CDifferentExpressedGene%5CFunctional_Enrichment%5CPathway%5CControl-VS-MT10.DEseq2_Method.htm#gene5%23gene5) | 489 (29.76%) | 3.277023E-10 | ko01100 |
| 6 | [Stilbenoid, diarylheptanoid and gingerol biosynthesis](../../../../F:%5C%E7%A7%91%E7%A0%94%5C%E5%AE%9E%E9%AA%8C%5C2016%5C%E8%A4%AA%E9%BB%91%E7%B4%A0%5C%E6%A0%80%E5%AD%90%5C%E8%BD%AC%E5%BD%95%E7%BB%84%5CTranscriptome_Denovo_Report%5CTranscriptome_Denovo_Report%5CBGI_result%5C5.Quantify%5CDifferentExpressedGene%5CFunctional_Enrichment%5CPathway%5CControl-VS-MT10.DEseq2_Method.htm#gene6%23gene6) | 29 (1.77%) | 9.488533E-08 | ko00945 |
| 7 | [Zeatin biosynthesis](../../../../F:%5C%E7%A7%91%E7%A0%94%5C%E5%AE%9E%E9%AA%8C%5C2016%5C%E8%A4%AA%E9%BB%91%E7%B4%A0%5C%E6%A0%80%E5%AD%90%5C%E8%BD%AC%E5%BD%95%E7%BB%84%5CTranscriptome_Denovo_Report%5CTranscriptome_Denovo_Report%5CBGI_result%5C5.Quantify%5CDifferentExpressedGene%5CFunctional_Enrichment%5CPathway%5CControl-VS-MT10.DEseq2_Method.htm#gene7%23gene7) | 17 (1.03%) | 1.092783E-04 | ko00908 |
| 8 | [Limonene and pinene degradation](../../../../F:%5C%E7%A7%91%E7%A0%94%5C%E5%AE%9E%E9%AA%8C%5C2016%5C%E8%A4%AA%E9%BB%91%E7%B4%A0%5C%E6%A0%80%E5%AD%90%5C%E8%BD%AC%E5%BD%95%E7%BB%84%5CTranscriptome_Denovo_Report%5CTranscriptome_Denovo_Report%5CBGI_result%5C5.Quantify%5CDifferentExpressedGene%5CFunctional_Enrichment%5CPathway%5CControl-VS-MT10.DEseq2_Method.htm#gene8%23gene8) | 23 (1.4%) | 1.554353E-04 | ko00903 |
| 9 | [Flavonoid biosynthesis](../../../../F:%5C%E7%A7%91%E7%A0%94%5C%E5%AE%9E%E9%AA%8C%5C2016%5C%E8%A4%AA%E9%BB%91%E7%B4%A0%5C%E6%A0%80%E5%AD%90%5C%E8%BD%AC%E5%BD%95%E7%BB%84%5CTranscriptome_Denovo_Report%5CTranscriptome_Denovo_Report%5CBGI_result%5C5.Quantify%5CDifferentExpressedGene%5CFunctional_Enrichment%5CPathway%5CControl-VS-MT10.DEseq2_Method.htm#gene9%23gene9) | 20 (1.22%) | 3.243925E-04 | ko00941 |
| 10 | [Glycerophospholipid metabolism](../../../../F:%5C%E7%A7%91%E7%A0%94%5C%E5%AE%9E%E9%AA%8C%5C2016%5C%E8%A4%AA%E9%BB%91%E7%B4%A0%5C%E6%A0%80%E5%AD%90%5C%E8%BD%AC%E5%BD%95%E7%BB%84%5CTranscriptome_Denovo_Report%5CTranscriptome_Denovo_Report%5CBGI_result%5C5.Quantify%5CDifferentExpressedGene%5CFunctional_Enrichment%5CPathway%5CControl-VS-MT10.DEseq2_Method.htm#gene10%23gene10) | 40 (2.43%) | 1.154952E-03 | ko00564 |
| 11 | [Isoflavonoid biosynthesis](../../../../F:%5C%E7%A7%91%E7%A0%94%5C%E5%AE%9E%E9%AA%8C%5C2016%5C%E8%A4%AA%E9%BB%91%E7%B4%A0%5C%E6%A0%80%E5%AD%90%5C%E8%BD%AC%E5%BD%95%E7%BB%84%5CTranscriptome_Denovo_Report%5CTranscriptome_Denovo_Report%5CBGI_result%5C5.Quantify%5CDifferentExpressedGene%5CFunctional_Enrichment%5CPathway%5CControl-VS-MT10.DEseq2_Method.htm#gene11%23gene11) | 12 (0.73%) | 1.755436E-03 | ko00943 |
| 12 | [Glycerolipid metabolism](../../../../F:%5C%E7%A7%91%E7%A0%94%5C%E5%AE%9E%E9%AA%8C%5C2016%5C%E8%A4%AA%E9%BB%91%E7%B4%A0%5C%E6%A0%80%E5%AD%90%5C%E8%BD%AC%E5%BD%95%E7%BB%84%5CTranscriptome_Denovo_Report%5CTranscriptome_Denovo_Report%5CBGI_result%5C5.Quantify%5CDifferentExpressedGene%5CFunctional_Enrichment%5CPathway%5CControl-VS-MT10.DEseq2_Method.htm#gene12%23gene12) | 30 (1.83%) | 5.012663E-03 | ko00561 |
| 13 | [Plant hormone signal transduction](../../../../F:%5C%E7%A7%91%E7%A0%94%5C%E5%AE%9E%E9%AA%8C%5C2016%5C%E8%A4%AA%E9%BB%91%E7%B4%A0%5C%E6%A0%80%E5%AD%90%5C%E8%BD%AC%E5%BD%95%E7%BB%84%5CTranscriptome_Denovo_Report%5CTranscriptome_Denovo_Report%5CBGI_result%5C5.Quantify%5CDifferentExpressedGene%5CFunctional_Enrichment%5CPathway%5CControl-VS-MT10.DEseq2_Method.htm#gene13%23gene13) | 56 (3.41%) | 5.534746E-03 | ko04075 |
| 14 | [Flavone and flavonol biosynthesis](../../../../F:%5C%E7%A7%91%E7%A0%94%5C%E5%AE%9E%E9%AA%8C%5C2016%5C%E8%A4%AA%E9%BB%91%E7%B4%A0%5C%E6%A0%80%E5%AD%90%5C%E8%BD%AC%E5%BD%95%E7%BB%84%5CTranscriptome_Denovo_Report%5CTranscriptome_Denovo_Report%5CBGI_result%5C5.Quantify%5CDifferentExpressedGene%5CFunctional_Enrichment%5CPathway%5CControl-VS-MT10.DEseq2_Method.htm#gene14%23gene14) | 9 (0.55%) | 5.534746E-03 | ko00944 |
| 15 | [Carotenoid biosynthesis](../../../../F:%5C%E7%A7%91%E7%A0%94%5C%E5%AE%9E%E9%AA%8C%5C2016%5C%E8%A4%AA%E9%BB%91%E7%B4%A0%5C%E6%A0%80%E5%AD%90%5C%E8%BD%AC%E5%BD%95%E7%BB%84%5CTranscriptome_Denovo_Report%5CTranscriptome_Denovo_Report%5CBGI_result%5C5.Quantify%5CDifferentExpressedGene%5CFunctional_Enrichment%5CPathway%5CControl-VS-MT10.DEseq2_Method.htm#gene15%23gene15) | 21 (1.28%) | 5.534746E-03 | ko00906 |
| 16 | [Galactose metabolism](../../../../F:%5C%E7%A7%91%E7%A0%94%5C%E5%AE%9E%E9%AA%8C%5C2016%5C%E8%A4%AA%E9%BB%91%E7%B4%A0%5C%E6%A0%80%E5%AD%90%5C%E8%BD%AC%E5%BD%95%E7%BB%84%5CTranscriptome_Denovo_Report%5CTranscriptome_Denovo_Report%5CBGI_result%5C5.Quantify%5CDifferentExpressedGene%5CFunctional_Enrichment%5CPathway%5CControl-VS-MT10.DEseq2_Method.htm#gene16%23gene16) | 29 (1.77%) | 1.169217E-02 | ko00052 |
| 17 | [Degradation of aromatic compounds](../../../../F:%5C%E7%A7%91%E7%A0%94%5C%E5%AE%9E%E9%AA%8C%5C2016%5C%E8%A4%AA%E9%BB%91%E7%B4%A0%5C%E6%A0%80%E5%AD%90%5C%E8%BD%AC%E5%BD%95%E7%BB%84%5CTranscriptome_Denovo_Report%5CTranscriptome_Denovo_Report%5CBGI_result%5C5.Quantify%5CDifferentExpressedGene%5CFunctional_Enrichment%5CPathway%5CControl-VS-MT10.DEseq2_Method.htm#gene17%23gene17) | 11 (0.67%) | 2.116737E-02 | ko01220 |
| 18 | [Pentose phosphate pathway](../../../../F:%5C%E7%A7%91%E7%A0%94%5C%E5%AE%9E%E9%AA%8C%5C2016%5C%E8%A4%AA%E9%BB%91%E7%B4%A0%5C%E6%A0%80%E5%AD%90%5C%E8%BD%AC%E5%BD%95%E7%BB%84%5CTranscriptome_Denovo_Report%5CTranscriptome_Denovo_Report%5CBGI_result%5C5.Quantify%5CDifferentExpressedGene%5CFunctional_Enrichment%5CPathway%5CControl-VS-MT10.DEseq2_Method.htm#gene18%23gene18) | 25 (1.52%) | 4.286004E-02 | ko00030 |
| 19 | [Plant-pathogen interaction](../../../../F:%5C%E7%A7%91%E7%A0%94%5C%E5%AE%9E%E9%AA%8C%5C2016%5C%E8%A4%AA%E9%BB%91%E7%B4%A0%5C%E6%A0%80%E5%AD%90%5C%E8%BD%AC%E5%BD%95%E7%BB%84%5CTranscriptome_Denovo_Report%5CTranscriptome_Denovo_Report%5CBGI_result%5C5.Quantify%5CDifferentExpressedGene%5CFunctional_Enrichment%5CPathway%5CControl-VS-MT10.DEseq2_Method.htm#gene19%23gene19) | 84 (5.11%) | 4.324229E-02 | ko04626 |
| 20 | [Ether lipid metabolism](../../../../F:%5C%E7%A7%91%E7%A0%94%5C%E5%AE%9E%E9%AA%8C%5C2016%5C%E8%A4%AA%E9%BB%91%E7%B4%A0%5C%E6%A0%80%E5%AD%90%5C%E8%BD%AC%E5%BD%95%E7%BB%84%5CTranscriptome_Denovo_Report%5CTranscriptome_Denovo_Report%5CBGI_result%5C5.Quantify%5CDifferentExpressedGene%5CFunctional_Enrichment%5CPathway%5CControl-VS-MT10.DEseq2_Method.htm#gene20%23gene20) | 12 (0.73%) | 4.324229E-02 | ko00565 |
| 21 | [Pentose and glucuronate interconversions](../../../../F:%5C%E7%A7%91%E7%A0%94%5C%E5%AE%9E%E9%AA%8C%5C2016%5C%E8%A4%AA%E9%BB%91%E7%B4%A0%5C%E6%A0%80%E5%AD%90%5C%E8%BD%AC%E5%BD%95%E7%BB%84%5CTranscriptome_Denovo_Report%5CTranscriptome_Denovo_Report%5CBGI_result%5C5.Quantify%5CDifferentExpressedGene%5CFunctional_Enrichment%5CPathway%5CControl-VS-MT10.DEseq2_Method.htm#gene21%23gene21) | 24 (1.46%) | 4.364652E-02 | ko00040 |

**Supplemental Table S4 Main DEGs included in six category pathways**.

| Gene ID | Gene name | log2 ratio (MT1.0/Control) | P-value | P-adj | Up/Down-  Regulation |
| --- | --- | --- | --- | --- | --- |
| CL1172.Contig5 | alpha-1,4-galacturonosyltransferase | 2.100936688 | 7.53E-05 | 0.001670422 | Up |
| CL4521.Contig5 | alpha-1,4-galacturonosyltransferase | 1.541845849 | 0.001150646 | 0.017800824 | Up |
| Unigene18196 | pectinesterase | 1.540740582 | 1.05E-22 | 1.48E-20 | Up |
| CL5464.Contig2 | UDP-glucuronate decarboxylase | 1.106605042 | 0.000446718 | 0.007922441 | Up |
| Unigene15705 | glucose-1-phosphate adenylyltransferase | 1.344448336 | 5.14E-07 | 1.79E-05 | Up |
| CL1888.Contig3 | glucose-1-phosphate adenylyltransferase | 1.301944744 | 4.99E-06 | 0.000144688 | Up |
| CL5614.Contig1 | starch synthase | 1.081619501 | 5.16E-05 | 0.001193058 | Up |
| CL6507.Contig3 | glycogen phosphorylase | 1.469998865 | 2.14E-13 | 1.59E-11 | Up |
| CL5611.Contig2 | glycogen debranching enzyme | 2.314784519 | 0.000154432 | 0.003127396 | Up |
| CL5611.Contig3 | glycogen debranching enzyme | 1.313834975 | 7.75E-05 | 0.001711548 | Up |
| CL6687.Contig6 | beta-galactosidase | 2.440583539 | 0.001537311 | 0.022707686 | Up |
| Unigene20868 | beta-galactosidase | 1.759274972 | 8.62E-06 | 0.000237898 | Up |
| Unigene23170 | aldehyde reductase | 2.367961841 | 0.001985295 | 0.028229987 | Up |
| Unigene390 | aldehyde reductase | 1.503022434 | 0.00044367 | 0.007875138 | Up |
| CL8094.Contig1 | aldehyde reductase | 1.380464242 | 1.43E-101 | 1.20E-98 | Up |
| CL2339.Contig1 | aldehyde reductase | 1.331815902 | 0.000653096 | 0.011060343 | Up |
| CL2415.Contig3 | stachyose synthetase | 1.20152299 | 0.002529916 | 0.034632385 | Up |
| CL2316.Contig1 | 6-phosphogluconolactonase | 1.174146486 | 0.000677634 | 0.011416173 | Up |
| CL7158.Contig3 | transaldolase | 1.212287807 | 9.06E-06 | 0.000248591 | Up |
| Unigene18302 | ribose 5-phosphate isomerase A | 1.441628339 | 2.01E-06 | 6.24E-05 | Up |
| CL266.Contig4 | phosphoglucomutase | 1.587974164 | 4.26E-05 | 0.001008978 | Up |
| CL1176.Contig8 | phosphoglucomutase | 1.144047369 | 2.34E-05 | 0.000587963 | Up |
| Unigene18109 | pectate lyase | 1.683107156 | 0.001371974 | 0.02060364 | Up |
| Unigene23170 | aldehyde reductase | 2.367961841 | 0.001985295 | 0.028229987 | Up |
| Unigene390 | aldehyde reductase | 1.503022434 | 0.00044367 | 0.007875138 | Up |
| CL153.Contig10 | D-ribulokinase | 2.704879891 | 0.000410714 | 0.00733745 | Up |
| Unigene18196 | pectinesterase | 1.540740582 | 1.05E-22 | 1.48E-20 | Up |
| CL9710.Contig1 | pectinesterase | 1.017804776 | 2.30E-05 | 0.000578787 | Up |
| CL5861.Contig2 | polygalacturonase | 1.153983111 | 0.002431873 | 0.033507786 | Up |
| Unigene12144 | aldehyde dehydrogenase (NAD+) | 1.298337202 | 0.001191902 | 0.018329652 | Up |
| CL1081.Contig8 | beta-glucosidase | 2.35805963 | 0.002185249 | 0.030625361 | Up |
| CL1081.Contig18 | beta-glucosidase | 2.310671434 | 0.002684082 | 0.036304959 | Up |
| CL1081.Contig13 | beta-glucosidase | 2.090760957 | 2.07E-05 | 0.000525497 | Up |
| CL1435.Contig8 | beta-glucosidase | 1.762693426 | 0.002391376 | 0.033065558 | Up |
| CL463.Contig9 | beta-glucosidase | 1.078160842 | 2.68E-08 | 1.14E-06 | Up |
| Unigene24886 | beta-glucosidase | 1.305087554 | 1.34E-10 | 7.64E-09 | Up |
| Unigene44600 | beta-glucosidase | 1.345768055 | 1.99E-10 | 1.12E-08 | Up |
| CL265.Contig15 | beta-glucosidase | 1.198943662 | 7.93E-08 | 3.14E-06 | Up |
| Unigene7026 | gamma-glutamyltranspeptidase / glutathione hydrolase | 1.02651891 | 1.25E-19 | 1.46E-17 | Up |
| CL3542.Contig2 | auxin influx carrier (AUX1 LAX family) | 1.436833127 | 4.78E-20 | 5.74E-18 | Up |
| Unigene21419 | SAUR family protein | 1.19644345 | 0.003921342 | 0.049495856 | Up |
| Unigene15143 | arabidopsis histidine kinase 2/3/4 (cytokinin receptor) | 1.426627224 | 0.000296042 | 0.005551653 | Up |
| CL8617.Contig1 | two-component response regulator ARR-A family | 2.01604949 | 0.000305226 | 0.005703887 | Up |
| CL600.Contig2 | two-component response regulator ARR-B family | 4.508654035 | 1.25E-10 | 7.18E-09 | Up |
| CL2259.Contig1 | two-component response regulator ARR-B family | 1.108421442 | 0.000602342 | 0.010306416 | Up |
| Unigene19027 | gibberellin receptor GID1 | 1.22351021 | 3.42E-11 | 2.08E-09 | Up |
| CL5033.Contig2 | phytochrome-interacting factor 4 | 1.502604572 | 2.58E-06 | 7.86E-05 | Up |
| CL7028.Contig2 | cytokinin trans-hydroxylase | 3.904750125 | 4.72E-08 | 1.94E-06 | Up |
| CL3489.Contig2 | cis-zeatin O-glucosyltransferase | 1.165415668 | 4.23E-05 | 0.001001631 | Up |
| CL3489.Contig6 | cis-zeatin O-glucosyltransferase | 1.07024293 | 2.56E-06 | 7.82E-05 | Up |
| CL3489.Contig11 | cis-zeatin O-glucosyltransferase | 1.076775457 | 5.76E-07 | 1.99E-05 | Up |
| CL6694.Contig1 | EIN3-binding F-box protein | -1.174345414 | 3.64E-08 | 1.52E-06 | Down |
| CL1771.Contig13 | ethylene-insensitive protein 3 | -1.513244259 | 0.000157105 | 0.003177636 | Down |
| CL1771.Contig5 | ethylene-insensitive | -1.258715833 | 2.58E-16 | 2.44E-14 | Down |
| Unigene21372 | ethylene-responsive transcription factor 1 | -1.229537105 | 1.71E-06 | 5.37E-05 | Down |
| Unigene10371 | transcription factor TGA | -2.534982513 | 1.02E-12 | 7.12E-11 | Down |
| Unigene7385 | transcription factor TGA | -1.911860815 | 0.000140984 | 0.002881187 | Down |
| CL3218.Contig1 | pathogenesis-related protein 1 | -1.786490666 | 0.00081915 | 0.013390132 | Down |
| CL3218.Contig4 | pathogenesis-related protein 1 | -1.258566765 | 0.003355896 | 0.043592444 | Down |
| Unigene12300 | protein brassinosteroid insensitive 1 | -1.812227819 | 0.000108768 | 0.002295379 | Down |
| CL1891.Contig3 | protein brassinosteroid insensitive 1 | -1.066748235 | 1.09E-09 | 5.61E-08 | Down |
| CL1891.Contig1 | protein brassinosteroid insensitive 1 | -1.012528023 | 6.40E-05 | 0.001443647 | Down |
| Unigene2092 | BIN2 protein brassinosteroid insensitive 2 | -2.274898022 | 0.003162925 | 0.041452077 | Down |
| CL9153.Contig1 | TCH4 xyloglucan: xyloglucosyl transferase | -1.682839822 | 0.000275442 | 0.005206222 | Down |
| Unigene19626 | jasmonate ZIM domain-containing protein | -4.618993323 | 1.93E-19 | 2.23E-17 | Down |
| CL9168.Contig1 | jasmonate ZIM | -2.114690264 | 8.75E-250 | 2.33E-246 | Down |
| CL4262.Contig3 | jasmonate ZIM | -1.264133579 | 8.31E-05 | 0.001821502 | Down |
| CL4262.Contig1 | jasmonate ZIM | -1.273091295 | 2.51E-22 | 3.46E-20 | Down |
| Unigene16440 | MYC2 transcription factor | -1.86114739 | 0.000832598 | 0.013569158 | Down |
| CL2540.Contig1 | MYC2 transcription factor | -1.673767929 | 5.21E-05 | 0.001202028 | Down |
| CL2478.Contig5 | MYC2 transcription factor | -1.126923805 | 8.90E-05 | 0.001932284 | Down |
| CL2478.Contig3 | MYC2 transcription factor | -1.030362337 | 0.000445387 | 0.007900523 | Down |
| CL25.Contig24 | glycerol-3-phosphate dehydrogenase (NAD+) | -3.714775516 | 5.90E-07 | 2.03E-05 | Down |
| CL25.Contig74 | glycerol-3-phosphate dehydrogenase (NAD+) | -2.517333413 | 0.001090921 | 0.017036153 | Down |
| CL4646.Contig3 | glycerol-3-phosphate dehydrogenase (NAD+) | -2.527157157 | 0.000593206 | 0.010170018 | Down |
| CL25.Contig36 | glycerol-3-phosphate dehydrogenase (NAD+) | -1.474731404 | 0.001223851 | 0.018726898 | Down |
| CL25.Contig71 | glycerol-3-phosphate dehydrogenase (NAD+) | -1.324745703 | 1.45E-05 | 0.000381955 | Down |
| CL25.Contig22 | glycerol-3-phosphate dehydrogenase (NAD+) | -1.28039507 | 4.96E-08 | 2.03E-06 | Down |
| CL25.Contig3 | glycerol-3-phosphate dehydrogenase (NAD+) | -1.280766709 | 7.49E-07 | 2.53E-05 | Down |
| CL25.Contig11 | glycerol-3-phosphate dehydrogenase (NAD+) | -1.249662113 | 7.46E-07 | 2.52E-05 | Down |
| CL25.Contig44 | glycerol-3-phosphate dehydrogenase (NAD+) | -1.225093548 | 1.86E-20 | 2.29E-18 | Down |
| CL25.Contig35 | glycerol-3-phosphate dehydrogenase (NAD+) | -1.158372455 | 1.53E-05 | 0.0003993 | Down |
| CL25.Contig12 | glycerol-3-phosphate dehydrogenase (NAD+) | -1.171751463 | 3.19E-07 | 1.15E-05 | Down |
| CL25.Contig75 | glycerol-3-phosphate dehydrogenase (NAD+) | -1.051766009 | 0.000296021 | 0.005551653 | Down |
| CL6835.Contig1 | lysophosphatidic acid acyltransferase | -2.203064422 | 0.003805825 | 0.048262845 | Down |
| CL7237.Contig1 | HRAS-like suppressor 3 | -4.81202233 | 0 | 0 | Down |
| CL7237.Contig2 | HRAS-like suppressor 3 | -4.72480453 | 0 | 0 | Down |
| Unigene3646 | HRAS-like suppressor 3 | -2.198524764 | 1.22E-23 | 1.79E-21 | Down |
| CL5923.Contig5 | glycerophosphodiester phosphodiesterase | -1.074931886 | 5.94E-14 | 4.69E-12 | Down |
| CL1548.Contig2 | choline kinase | -1.17959151 | 1.58E-42 | 4.68E-40 | Down |
| CL2204.Contig2 | aldehyde dehydrogenase (NAD+) | -2.849819752 | 4.30E-08 | 1.78E-06 | Down |
| CL4268.Contig4 | glycerol-3-phosphate O-acyltransferase 1/2 | -3.215400861 | 1.12E-06 | 3.67E-05 | Down |
| CL2764.Contig1 | glycerol-3-phosphate O-acyltransferase 1/2 | -1.820224258 | 1.18E-24 | 1.83E-22 | Down |
| CL6050.Contig1 | glycerol-3-phosphate O-acyltransferase 1/2 | -1.319287498 | 9.54E-08 | 3.73E-06 | Down |
| CL330.Contig4 | glycerol-3-phosphate O-acyltransferase 1/2 | -1.180452224 | 9.30E-12 | 6.02E-10 | Down |
| CL2764.Contig8 | glycerol-3-phosphate O-acyltransferase 1/2 | -1.055715917 | 0.000402537 | 0.007210063 | Down |
| CL7451.Contig1 | 1-acyl-sn-glycerol-3-phosphate acyltransferase | -1.614012597 | 1.02E-95 | 8.11E-93 | Down |
| CL397.Contig3 | diacylglycerol kinase (ATP) | -1.315944913 | 0.000443189 | 0.00787118 | Down |
| CL6169.Contig1 | 1,2-diacylglycerol 3-beta-galactosyltransferase | -1.547627896 | 6.07E-06 | 0.000173382 | Down |
| CL6169.Contig3 | 1,2-diacylglycerol 3-beta-galactosyltransferase | -1.093823242 | 8.54E-33 | 1.89E-30 | Down |
| CL1392.Contig2 | diacylglycerol O-acyltransferase | -1.427514524 | 7.23E-37 | 1.84E-34 | Down |
| Unigene12951 | phospholipid: diacylglycerol acyltransferase | -2.594515249 | 7.96E-05 | 0.001753333 | Down |
| Unigene765 | platelet-activating factor acetylhydrolase | -4.287188634 | 8.71E-197 | 1.71E-193 | Down |
| CL7451.Contig6 | lysophosphatidylcholine acyltransferase | -2.402330291 | 0.000961045 | 0.015318959 | Down |
| CL7451.Contig2 | lysophosphatidylcholine acyltransferase | -1.742129966 | 1.22E-08 | 5.46E-07 | Down |
| CL7451.Contig4 | lysophosphatidylcholine acyltransferase | -1.511980129 | 7.41E-35 | 1.76E-32 | Down |
| CL7451.Contig5 | lysophosphatidylcholine acyltransferase | -1.394673157 | 1.53E-15 | 1.37E-13 | Down |
| CL7451.Contig3 | lysophosphatidylcholine acyltransferase | -1.324589685 | 8.21E-13 | 5.77E-11 | Down |
| Unigene22398 | 1-acylglycerone phosphate reductase | -2.252488897 | 0.000497048 | 0.008691916 | Down |
| CL5182.Contig1 | ethanolaminephosphotransferase | -1.017258132 | 6.02E-18 | 6.33E-16 | Down |
| CL66.Contig3 | phospholipase D1/2 | -1.690411152 | 0.000200316 | 0.003938835 | Down |
| CL66.Contig25 | phospholipase D1/2 | -1.01955967 | 6.35E-05 | 0.001435557 | Down |
| Unigene28270 | flavonoid 3'-monooxygenase | -2.997389556 | 7.42E-05 | 0.001647595 | Down |
| CL9245.Contig2 | shikimate O-hydroxycinnamoyltransferase | -2.529500704 | 7.81E-14 | 6.08E-12 | Down |
| CL1505.Contig4 | shikimate O-hydroxycinnamoyltransferase | -2.333245554 | 3.06E-05 | 0.000749593 | Down |
| CL9197.Contig1 | shikimate O-hydroxycinnamoyltransferase | -1.379481912 | 2.81E-07 | 1.02E-05 | Down |
| CL3275.Contig2 | shikimate O-hydroxycinnamoyltransferase | -1.151085853 | 7.30E-14 | 5.70E-12 | Down |
| Unigene27090 | shikimate O-hydroxycinnamoyltransferase | -1.047835414 | 0.000817626 | 0.013372623 | Down |
| CL3878.Contig1 | flavonol synthase | -2.101149646 | 3.81E-11 | 2.31E-09 | Down |
| CL8916.Contig2 | flavonoid 6-hydroxylase | -2.401647266 | 2.34E-06 | 7.19E-05 | Down |
| CL5940.Contig1 | 2-hydroxyisoflavanone dehydratase | -1.132655914 | 7.22E-05 | 0.001607696 | Down |
| CL3123.Contig1 | isoflavone 2'-hydroxylase | -3.079258092 | 7.06E-82 | 4.49E-79 | Down |
| Unigene7816 | isoflavone 2'-hydroxylase | -2.801690539 | 1.18E-13 | 8.97E-12 | Down |
| CL3123.Contig5 | isoflavone 2'-hydroxylase | -1.993877358 | 4.35E-79 | 2.68E-76 | Down |
| Unigene7276 | isoflavone 2'-hydroxylase | -1.922488357 | 4.02E-28 | 7.17E-26 | Down |
| CL3123.Contig6 | isoflavone 2'-hydroxylase | -1.685058127 | 2.08E-13 | 1.55E-11 | Down |
| CL3123.Contig7 | isoflavone 2'-hydroxylase | -1.291676516 | 7.26E-14 | 5.67E-12 | Down |
| Unigene17741 | isoflavone 2'-hydroxylase | -1.273343572 | 1.62E-07 | 6.09E-06 | Down |
| CL9740.Contig2 | isoflavone 2'-hydroxylase | -1.175277785 | 1.12E-17 | 1.16E-15 | Down |
| Unigene5707 | flavonoid 3',5'-hydroxylase | -1.225959813 | 0.000182242 | 0.003623087 | Down |
| CL804.Contig1 | flavonoid 3'-monooxygenase | -3.102128174 | 2.52E-210 | 5.47E-207 | Down |
| CL295.Contig8 | flavonoid 3'-monooxygenase | -2.625413047 | 0.000612231 | 0.01044534 | Down |
| CL7024.Contig1 | flavonoid 3'-monooxygenase | -2.154180885 | 0.001067431 | 0.016745772 | Down |
| Unigene16046 | isoflavone 7-O-glucoside-6''-O-malonyltransferase | -1.202050725 | 4.79E-18 | 5.09E-16 | Down |
| Unigene16036 | zeta-carotene desaturase | -3.420383925 | 3.46E-07 | 1.24E-05 | Down |
| CL9294.Contig1 | zeta-carotene desaturase | -1.281631954 | 7.49E-153 | 1.03E-149 | Down |
| CL1480.Contig36 | prolycopene isomerase | -4.998617038 | 2.34E-13 | 1.73E-11 | Down |
| CL1480.Contig49 | prolycopene isomerase | -4.18017793 | 6.68E-09 | 3.10E-07 | Down |
| CL1480.Contig1 | prolycopene isomerase | -2.415756671 | 0.001712354 | 0.024888467 | Down |
| CL1480.Contig28 | prolycopene isomerase | -1.933768766 | 8.38E-07 | 2.81E-05 | Down |
| CL7164.Contig5 | zeaxanthin epoxidase | -1.244694136 | 0.000488442 | 0.008566809 | Down |
| CL7164.Contig2 | zeaxanthin epoxidase | -1.249744859 | 0.002332215 | 0.032377549 | Down |
| CL6344.Contig1 | 9-cis-epoxycarotenoid dioxygenase | -1.601922395 | 6.93E-22 | 9.30E-20 | Down |
| CL6344.Contig2 | 9-cis-epoxycarotenoid dioxygenase | -1.130465185 | 0.000779633 | 0.012845286 | Down |
| Unigene719 | abscisate beta-glucosyltransferase | -1.822978778 | 1.84E-24 | 2.83E-22 | Down |
| CL3322.Contig1 | abscisate beta-glucosyltransferase | -1.396067937 | 6.41E-129 | 7.16E-126 | Down |
| CL3322.Contig2 | abscisate beta-glucosyltransferase | -1.049310851 | 1.06E-23 | 1.56E-21 | Down |
